# *Helicobacter pylori*-induced gastric pathology: insights from *in vivo* and *ex vivo* models

**DOI:** 10.1242/dmm.027649

**Published:** 2017-02-01

**Authors:** Michael D. Burkitt, Carrie A. Duckworth, Jonathan M. Williams, D. Mark Pritchard

**Affiliations:** 1 Gastroenterology Research Unit, Department of Cellular and Molecular Physiology, Institute of Translational Medicine, University of Liverpool, Liverpool L69 3GE,UK; 2 Pathology and Pathogen Biology, Royal Veterinary College, North Mymms AL9 7TA, UK

**Keywords:** *Helicobacter*, Gastric cancer, Peptic ulcer disease, MALT lymphoma, Organoid, Gastroid

## Abstract

Gastric colonization with *Helicobacter pylori* induces diverse human pathological conditions, including superficial gastritis, peptic ulcer disease, mucosa-associated lymphoid tissue (MALT) lymphoma, and gastric adenocarcinoma and its precursors. The treatment of these conditions often relies on the eradication of *H. pylori*, an intervention that is increasingly difficult to achieve and that does not prevent disease progression in some contexts. There is, therefore, a pressing need to develop new experimental models of *H. pylori*-associated gastric pathology to support novel drug development in this field. Here, we review the current status of *in vivo* and *ex vivo* models of gastric *H. pylori* colonization, and of *Helicobacter*-induced gastric pathology, focusing on models of gastric pathology induced by *H. pylori*, *Helicobacter felis* and *Helicobacter suis* in rodents and large animals. We also discuss the more recent development of gastric organoid cultures from murine and human gastric tissue, as well as from human pluripotent stem cells, and the outcomes of *H. pylori* infection in these systems.

## Abstract (precis)

**Summary:** *Helicobacter pylori*-induced gastric pathology remains a common cause of morbidity and mortality. This Review discusses the animal models of these conditions, together with recently described *ex vivo* culture models.

## Introduction

**Fig. 1.**
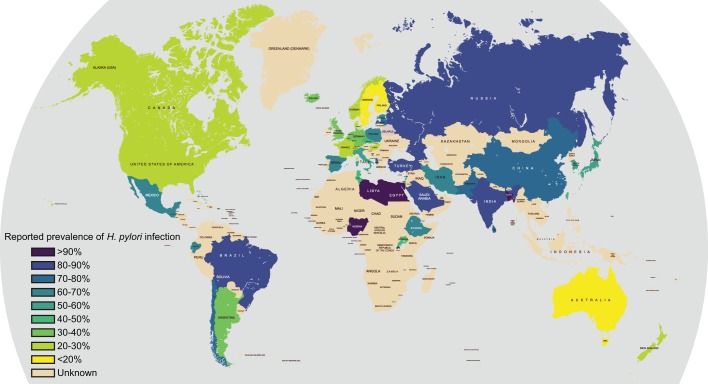
**Worldwide prevalence of *Helicobacter pylori* infection.** The map shows the prevalence of *H. pylori* infection in different parts of the world. Note, the particularly high prevalence in sub-Saharan Africa, Latin America and the Middle East. Australasia, Switzerland, and more generally North America and Western Europe have the lowest incidence of *H. pylori* infection. Data derived from Asfeldt et al., 2008; Ben Mansour et al., 2016; Laszewicz et al., 2014; Luzza et al., 2014; McDonald et al., 2015; Peleteiro et al., 2014; Saltanova, 2001; Sanchez Ceballos et al., 2007; van Blankenstein et al., 2013.

*Helicobacter pylori* is a bacterium that grows in close association with the lining of the stomach and is associated with various human gastric diseases; it causes significant morbidity and mortality worldwide. Globally, there are wide variations in the reported prevalence of *H. pylori* (Fig. 1), with particularly high levels observed in South America, sub-Saharan Africa and the Middle East (Asfeldt et al., 2008; Ben Mansour et al., 2016; Laszewicz et al., 2014; Luzza et al., 2014; McDonald et al., 2015; Peleteiro et al., 2014; Saltanova, 2001; Sanchez Ceballos et al., 2007; van Blankenstein et al., 2013).

Many publications have promoted the concept of ‘the African Enigma’ (Holcombe, 1992), owing to the reporting of fewer cases of peptic ulceration than expected in this continent. However, recent studies suggest that gastric pathology is endemic where *H. pylori* is endemic (Agha and Graham, 2005). They also suggest that the geographical distribution of gastric disease and its predominant form relates more to other co-factors, such as *H. pylori* virulence properties, food preservation methods, diet and host genetics (Graham et al., 2009; Kodaman et al., 2014).

One of the principal confounders of studies examining the impact of *H. pylori* infection on gastric health in diverse populations is the inequality of access to healthcare systems globally. Diagnosing *Helicobacter* infection relies on one of four tests: endoscopy with biopsy; ^13^Carbon-hydrogen breath test; faecal antigen testing; or serological detection of an anti-*H. pylori* antibody. These tests are relatively expensive and their availability is limited, particularly in developing countries where the highest *H. pylori* prevalence has been reported.

For many *H. pylori*-associated conditions, the most effective clinical intervention is to eradicate the infection using a combination of acid-suppressing medication and antibiotics. However, this strategy is becoming increasingly difficult to sustain because of the emergence of antibiotic-resistant *H. pylori* strains. Moreover, in some clinical circumstances, *H. pylori* eradication is ineffective at preventing disease progression. There is, therefore, an unmet need to develop new drugs, both to eradicate *H. pylori* more effectively and to offer alternative strategies where eradication of infection does not prevent the progression of gastric pathology.

To achieve this, we need to improve our understanding of the molecular events that lead to *H. pylori*-induced gastric pathology, and this requires experimental models. Here, we review the currently available *in vivo* and *ex vivo* models of *Helicobacter*-induced pathology, and describe the spectrum of pathology induced by infection with *H. pylori*, *Helicobacter felis* and *Helicobacter suis*. The *in vivo* models discussed here span rodent and larger animal models, including cat, dog, pig and non-human primate models, whilst the *ex vivo* models derive from mouse and human gastric mucosa and from pluripotent stem cells. It is particularly timely to review these *ex vivo* models because of the recent development of long-lived *ex vivo* cultures of untransformed gastric epithelium (Barker et al., 2010). These offer an important adjunct to the more established animal models of gastric carcinogenesis, and make it likely that future mechanistic studies of gastric disease will incorporate elements of both *in vivo* and *ex vivo* experimentation.

## *Helicobacter pylori*: an overview

**Fig. 2.**
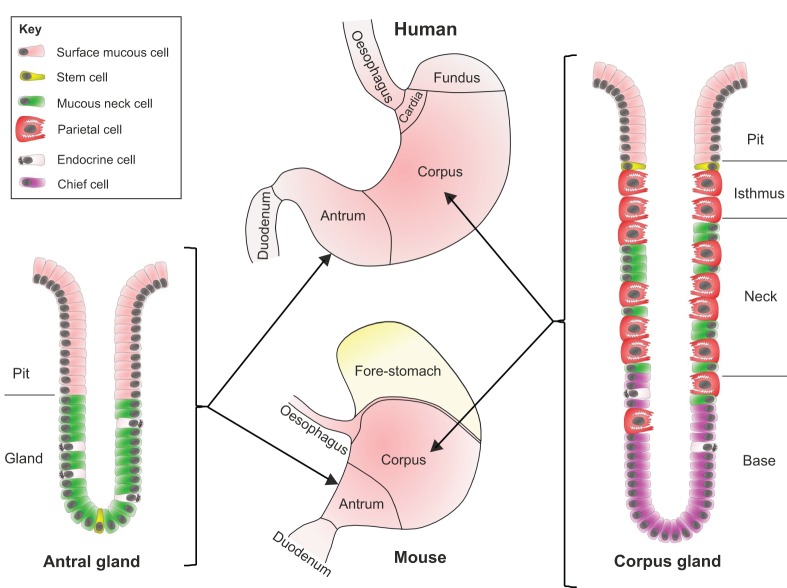
**The anatomy of the human and mouse stomach.** A schematic of the anatomy of the human and mouse stomach and the structure of gastric glands. Two types of columnar mucosa line the human stomach: the antrum is lined with antral glands, whilst the corpus and fundus are lined with deeper oxyntic, or corpus glands (see Glossary, Box 1). The murine stomach has areas that are analogous to the human stomach, including antral and corpus glands, and it also has a forestomach lined with squamous epithelium. Stem cells that reside at the base of the gland generate the antral gland. Following asymmetric cell division in the stem cell zone, daughter cells migrate upwards towards the gastric lumen and differentiate into mucous neck, surface mucous and endocrine cells. In corpus glands, the stem cell niche is located at the isthmus of the gland. Cells migrate upwards from the stem cell zone and differentiate into surface mucous cells. Other cells migrate down the gland and differentiate into acid-secreting parietal cells, endocrine cells, or zymogen-secreting (see Glossary, Box 1) chief cells.

The gastric microenvironment (Fig. 2) is hostile to commensal bacteria because of its low partial oxygen pressure, and the presence of high concentrations of gastric acid and digestive enzymes. *H. pylori* is a Gram-negative, spiral rod-shaped bacterium that has evolved to survive in this environment. Its adaptations to these conditions include an ability to tolerate a microaerophilic environment (see Glossary, Box 1), the expression of a urease enzyme that modulates the bacterial microenvironment by raising pH, and flagellae that provide motility, allowing *H. pylori* to access the deep mucous layer of the stomach wall, thereby utilizing the host mucosal defences to develop a survivable niche.

**Box 1. Glossary**

**Anthroponosis:** Infectious disease transmitted to a non-human species from humans.

**CagA:** *Helicobacter pylori* virulence factor. The secreted component of a type IV secretion system (the cag pathogenicity island) that is associated with more severe gastric pathology.

**Correa model:** A model of gastric carcinogenesis first proposed by Pelayo Correa et al. in 1975, describing the development of gastritis, gastric atrophy, gastric intestinal-type metaplasia, dysplasia and gastric intestinal-type adenocarcinoma.

**Dysplasia:** Replacement of normal gastric mucosa with structurally abnormal tissue with potentially abnormal proliferation, disordered arrangement of cells within the tissue, and structurally abnormal cells.

**Gastric organoid/gastroid:** An organoid generated from gastric epithelial stem cells.

**Gastrin:** A hormone produced in the stomach that stimulates the production of acid by parietal cells, mainly through its interaction with histamine-secreting ECL cells. In addition to its role in stimulating gastric acid secretion, gastrin is also a growth factor and stimulates gastric epithelial cell proliferation.

**Gastritis:** Inflammation of the epithelial lining of the stomach.

**Hypergastrinemia:** An elevated level of the hormone gastrin in the bloodstream. This is observed particularly in the context of gastric pre-neoplasia, where the growth factor function of gastrin is thought to promote increased epithelial cell proliferation.

**Metaplasia:** Replacement of one differentiated epithelial tissue by another, which is abnormal for that anatomical site.

**Microaerophilic:** A term that describes a bacterium adapted to survive in an environment with reduced partial oxygen pressure.

**Organoid:** A primary epithelial cell culture (usually 3D) that contains healthy, proliferating epithelial stem cells, which can be expanded and passaged multiple times, and generates daughter cells representative of all lineages generated by the stem cell *in vivo*.

**Oxyntic gland:** An acid-secreting gland that consists of an epithelial monolayer with a proliferative stem cell zone towards the upper third of the gland. Asymmetric proliferation in this region generates daughter cells that migrate both up and down the gland and differentiate into mucous neck cells, parietal cells, chief cells and enteroendocrine cells.

**Pars oesophagea:** A small area of non-glandular squamous mucosa near the oesophageal opening present in the stomachs of some animals that is analogous to the oesophageal mucosa in humans.

**Polymorphonuclear leukocyte infiltration:** The recruitment of multi-lobulated white blood cells (including neutrophils, basophils and eosinophils) into an epithelial tissue. Indicative of active inflammation in the tissue.

**Zoonosis:** Infectious disease in a human transmitted from an animal host.

**Zymogen:** The inactive form of a digestive enzyme, for example pepsinogen, which is an inactive form of the proteolytic enzyme, pepsin. Pepsinogen is activated by gastric acid secreted by parietal cells.

The transmission of *H. pylori* infection is considered to occur through an oro-oral or faeco-oral route. Data from families indicates that vertical transmission from parent to child is a common transmission route. A recent phylogenetic study in an Iranian population examined transmission by DNA fingerprinting of *H. pylori* 16S ribosomal subunit DNA obtained from faecal samples. This assay detected *H. pylori* DNA in 26 of 30 cases, and demonstrated the vertical transmission of *H. pylori* in 46.1% of families, with 38.4% of cases being colonized with an *H. pylori* strain phylogenetically identical to their mother's strain, and 7.7% with a strain identical to that of their father (Mamishi et al., 2016). These findings agree with similar studies performed in other populations (Konno et al., 2005, 2008; McMillan et al., 2011; Roma-Giannikou et al., 2003), and are supported by animal studies. For example, one study reported that in an *H. pylori*-infected cat colony, kittens were passively colonized by *H. pylori* over the first 14 weeks of life (Straubinger et al., 2003).

The global prevalence of *H. pylori* infection in humans is estimated to be 50%. The association of *H. pylori* with humans is longstanding, with phylogenetic studies suggesting that *H. pylori* strains have co-evolved with human populations since before the migration of early humans from Africa 58,000 years ago (Falush et al., 2003; Linz et al., 2007). Below, we discuss the consequences of *H. pylori* infection for human health, to establish the types of pathology that need to be modelled in the laboratory.

## *Helicobacter*-induced gastric pathology in humans

Chronic infection with *H. pylori* is strongly associated with gastric pathology, including chronic active gastritis (see Glossary, Box 1), peptic ulcer disease, gastric adenocarcinoma and gastric extranodal marginal zone lymphoma of mucosa-associated lymphoid tissue type (MALT lymphoma). Of these outcomes, the most significant in terms of mortality is gastric adenocarcinoma. Recent meta-analyses suggest that the relative risk of developing gastric cancer is 2- to 3-times higher for people infected with *H. pylori* than for those without infection (Danesh, 1999; Helicobacter and Cancer Collaborative Group, 2001). Understanding these different pathological conditions is important for understanding how faithfully the available models recapitulate the clinical features of *H. pylori* pathology.

### Superficial gastritis

The commonest outcome of *H. pylori* infection is gastritis. Acute gastritis has rarely been described in humans, but has been reported in the context of experimentalists being exposed to *H. pylori* either accidentally (Sobala et al., 1991) or in a deliberate attempt to induce gastric pathology (Marshall et al., 1985; Morris and Nicholson, 1987; Morris et al., 1991). In these cases, the infected individuals reported symptoms and underwent endoscopic assessment with biopsy of the inflamed gastric mucosa. The early stages of disease are marked by the presence of a polymorphonuclear leukocyte infiltrate (see Glossary, Box 1) in the gastric mucosa and a transient reduction in gastric acid output.

In the cases of Marshall et al. (1985) and Morris and Nicholson (1987), *H. pylori* eradication therapy was prescribed. This was effective in eradicating *H. pylori* from the gastric mucosa, and led to the complete resolution of symptoms and of gastric histological abnormalities. In the case of Sobala et al. (1991) symptoms and signs resolved spontaneously, and repeat endoscopy demonstrated low levels of *Helicobacter* colonizing the gastric antrum, together with an increase in lymphocytes within the gastric mucosa. These histological changes correlated with IgM and IgG seroconversion for *H. pylori*, which are typical for chronic, superficial *H. pylori* gastritis. This is the most prevalent *H. pylori*-induced gastric pathology worldwide (Campbell et al., 2001; Filipe et al., 1995; Potet et al., 1993).

### Peptic ulcer disease

Individuals colonized with *H. pylori* have a 6.8-fold [95% confidence interval (CI), 2.9-16.1] higher risk of developing peptic ulcer disease (PUD) than those not exposed to this infection (Li et al., 2010). In line with this, the reduced incidence of *H. pylori* infection worldwide has coincided with a reduction in PUD (Groenen et al., 2009). In contrast to the 1980s, when the association of *H. pylori* and PUD was first established (Graham, 1989), individuals presenting with this disease are now less likely to be colonized with *H. pylori*; more often, their condition is linked to non-steroidal anti-inflammatory drug use or to low-dose aspirin (Musumba et al., 2012; Sung et al., 2009).

*H. pylori*-induced peptic ulceration occurs in the context of pre-existing chronic superficial gastritis, but is associated with increased gastric acid secretion and a T helper 1 (Th1) polarized immune response, compared with individuals with isolated superficial gastritis (D'Elios et al., 1997; Shimada et al., 2002).

Frequently, individuals with PUD exhibit antral predominant gastritis, which leads to enhanced gastrin secretion (see Glossary, Box 1). In turn, this stimulates the parietal cells of the gastric corpus (Fig. 2) to secrete more acid (McColl et al., 1997), leading to mucosal ulceration. Eradication of *H. pylori* is reportedly sufficient to suppress excess gastrin secretion (McColl et al., 1991), which is an important component of the healing process of *H. pylori*-associated peptic ulcers.

### Gastric adenocarcinoma and its precursor lesions

In 2012, gastric cancer was the fifth commonest malignancy worldwide, and the third commonest cause of cancer-related death, with over 720,000 deaths worldwide caused by the disease (Ferlay et al., 2013). *H. pylori* colonization is the single biggest risk factor for gastric carcinogenesis and is a risk factor in at least 80% of cases of gastric cancer (Graham, 2015). However, as only a very small percentage of people infected with *H. pylori* go on to develop gastric cancer, understanding why those individuals do so is a key aim of future studies in this field.

**Fig. 3.**
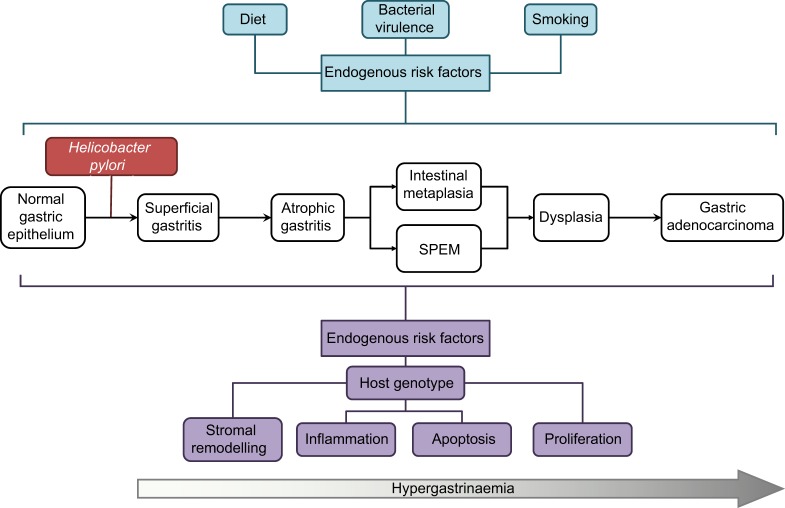
***H. pylori* infection and progression to gastric cancer.** A schematic demonstrating the pathological progression of *H. pylori*-induced gastric pre-neoplasia, and highlighting endogenous risk factors for progression towards gastric cancer. SPEM, spasmolytic polypeptide-expressing metaplasia.

Other risk factors linked to gastric cancer (Fig. 3) fall into two main groups. The first consists of potentially modifiable exogenous risk factors, such as dietary salt and nitrosamine intake (Jakszyn et al., 2006; Mendez et al., 2007; Wang et al., 2009), *H. pylori* virulence factors (Yamaoka, 2010), non-*Helicobacter* gastric microbiota (Dicksved et al., 2009; Lofgren et al., 2011) and smoking status (La et al., 2009). The second group consists of unalterable host genetic, or intrinsic, risk factors. Amongst these genetic factors are polymorphisms at loci encoding cytokines and their receptors (Persson et al., 2011), stromal remodelling proteins, such as matrix metalloproteinases (Tang et al., 2008), and prostate stem cell antigen (PSCA), which in the context of gastric pathology, acts as a tumour suppressor gene (Garcia-Gonzalez et al., 2015; Ichikawa et al., 2015; Mou et al., 2015).

The development of gastric cancer occurs through a stereotypical pathological pathway (Fig. 3, and Glossary, Box 1), which was first proposed well before the identification of *H. pylori* (Correa et al., 1975). Over the course of several decades, some individuals with chronic superficial gastritis develop gastric atrophy, characterized by the patchy loss of parietal cells in the gastric corpus mucosa. This decreases gastric acid secretion, leading to higher intraluminal pH, decreased somatostatin secretion and consequent gastrin secretion. In addition to stimulating acid secretion from parietal cells, gastrin also enhances proliferation in the gastric epithelial stem cell zone (Burkitt et al., 2009), leading to an increase in epithelial cell turnover.

A proportion of people with established gastric atrophy develop intestinal-type metaplasia (see Glossary, Box 1) of the gastric mucosa over time, where oxyntic glands (see Glossary, Box 1) are replaced by CDX2 (caudal-type homeobox 2)-expressing glandular units, which are morphologically similar to the intestinal crypt. Intestinal metaplasia in the stomach is linked to gastric dysplasia (see Glossary, Box 1); up to 20% of affected individuals with intestinal metaplasia have concurrent dysplasia (den Hoed et al., 2011). Gastric epithelial dysplasia is associated with an at least 10-fold increased risk of developing gastric cancer (You et al., 1999), but it has been difficult to represent this risk accurately from population-based studies.

Several studies have assessed the strategy of testing for, and eradicating, *H. pylori* in populations at a high risk of developing gastric cancer. Unfortunately, a recent well-designed meta-analysis confirmed a relatively poor outcome for this strategy. The eradication of *H. pylori* in this study reduced the risk of developing gastric cancer by about one-third [odds ratio (OR), 0.66; 95% CI, 0.46-0.95] (Ford et al., 2014). However, when individuals with pre-existing pre-neoplastic gastric pathology (defined as the presence of gastric atrophy, intestinal metaplasia or dysplasia) were considered, there was no evidence that eradication of *H. pylori* decreased the risk of gastric cancer (OR, 0.86; 95% CI, 0.47-1.59). For this highest risk group, therefore, there are currently no effective therapeutic strategies.

### MALT lymphoma

Gastric extranodal marginal zone lymphomas of mucosa-associated lymphoid tissue (MALT lymphomas) are B-cell lymphomas that develop within the mucosa-associated lymphoid tissue of the stomach. The incidence rate of gastric MALT lymphoma in the USA was estimated to be 3.8 in 1,000,000 individuals between 2001 and 2009, making it a rare outcome of *H. pylori* infection (Khalil et al., 2014). In the only published systematic review of this condition, 79% of 1844 reported cases of MALT lymphoma were associated with *H. pylori* infection (Asenjo and Gisbert, 2007; Gisbert and Calvet, 2011).

As with other haematological malignancies, characteristic cytogenetic profiles have been described for MALT lymphoma. Amongst the most well characterized is the formation of the *MALT1-API2* fusion oncogene by the t (11:18) translocation. This results in the expression of *API2* (encoding the cellular inhibitor of apoptosis 2) under the control of the *MALT1* promoter (Rosebeck et al., 2011). *MALT1* encodes mucosa-associated lymphoid tissue lymphoma translocation protein 1, which is essential for the activation and proliferation of T- and B-lymphocytes, and also plays a fundamental role in NF-κB activation. One of the downstream effects of this fusion protein is enhanced cleavage of NIK (NF-κB-inducing kinase), which is a critical regulator of alternative pathway NF-κB signalling (Merga et al., 2016).

## Human infections with other *Helicobacter* species

Whilst infection of the gastric mucosa with *H. pylori* is by far the most frequently observed gastric infection in humans, non-*H. pylori Helicobacter* (NHPH) species* * infections of human hosts have been identified since at least the mid-1990s. The identification of these organisms remains a challenge, and relies on molecular microbiological techniques that are not routinely available.

Although NHPH infections are frequently reported to occur in association with gastritis, different studies have yielded conflicting results as to their significance (Flahou et al., 2013; Liu et al., 2015). Understanding the contribution of NHPH species to gastritis is further complicated by the occurrence of mixed NHPH infections, by the heterogeneity of NHPH strains, by the nomenclature of these species, and the inability to cultivate many of them. For example, long spiral-shaped bacteria that were first recognized as microscopically different from *H. pylori* were isolated from human gastric biopsies and named *Gastrospirillum hominis* (McNulty et al., 1989). These organisms were subsequently reclassified as *Helicobacter heilmannii* based on 16S RNA analysis, of which there are at least two strains (Heilmann and Borchard, 1991). Many species of spiral-shaped NHPHs have since been found in the stomachs of animals (discussed in more detail below).

The most robust data for the pathogenicity of NHPH involve MALT lymphoma formation. *Helicobacter heilmannii-*associated MALT lymphoma was first described in 2000. Following this, a large study examining the prevalence of MALT lymphoma in 263,680 *H. pylori*-infected and 543 NHPH-infected people, demonstrated an odds ratio of 2.2 (95% CI, 1.1-4.5) for developing gastric MALT lymphoma in individuals infected with NHPH rather than with *H. pylori* (Stolte et al., 2002). This organism has also been described in association with individuals with chronic gastritis (Heilmann and Borchard, 1991). These observations suggest that NHPHs play a role in the development of human disease, and might in some cases be as pathogenic as *H. pylori*. The additional challenge of identifying these organisms suggests that there could be a group of individuals with gastric pathology due to unidentified NHPHs, representing an unmet clinical need.

## Naturally occurring gastric *Helicobacter* infections in non-human mammals

Whilst the association of *H. pylori* with humans has been extensively studied over the past 40 years, significantly less has been published on the association of *H. pylori*, or other *Helicobacter* species with different mammalian hosts. The data that are available suggest a spectrum of pathogenicity for different *Helicobacter* species, as well as a spectrum of susceptibility for gastric pathology in different host organisms. A better understanding of these comparative biological responses may, in the future, offer insights into the mechanisms that underlie human disease.

Forty-five species of *Helicobacter* have been detected by PCR in faecal samples from 150 vertebrate species, demonstrating their colonization of the digestive system of a wide range of domesticated and wild vertebrate species (Schrenzel et al., 2010). Spontaneous gastric colonization by NHPH has also been demonstrated in several mammalian species (Table 1), leading to speculation that all mammals harbour one or more *Helicobacter* species as part of their natural gastric flora (Brown et al., 2007).

**Table 1.**
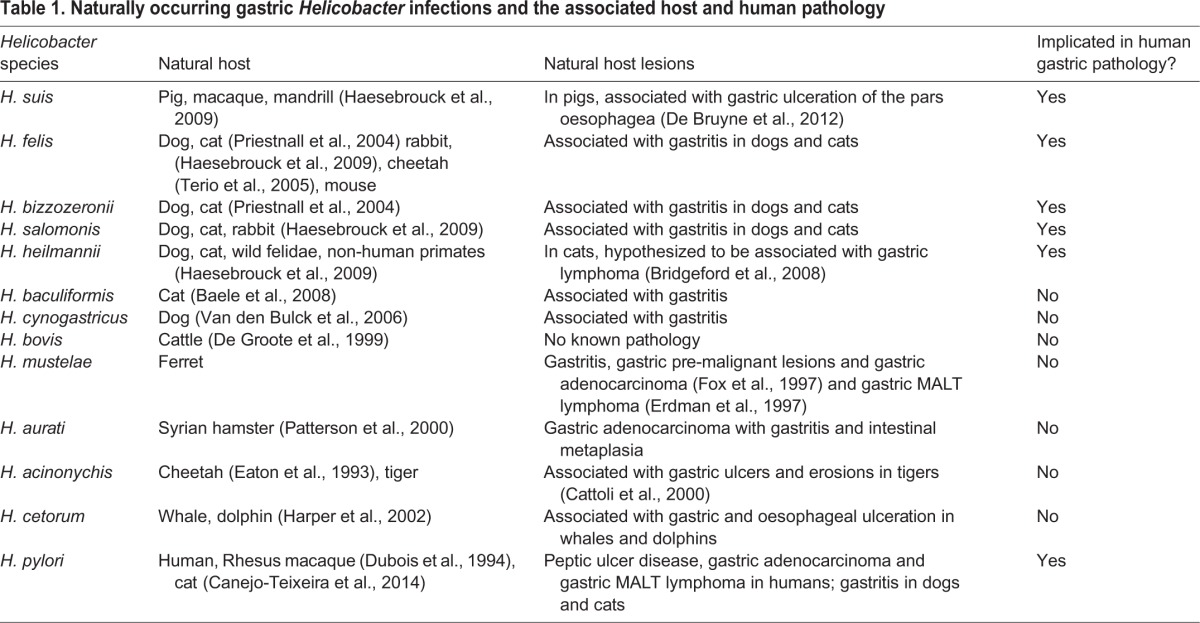
**Naturally occurring gastric *Helicobacter* infections and the associated host and human pathology**

When examining the evidence for NHPH-induced gastric lesions in animal species, various factors need to be considered, such as differences in gastric morphology, and in the distribution and site of gastric *Helicobacter* strains among some animal species and humans. Furthermore, the sequential pathological lesions that lead to adenocarcinoma formation in humans, as described by Correa's model (see Glossary, Box 1), have rarely been established in clinical veterinary species (Amorim et al., 2016), in which gastric cancer is rare. Exceptions to this include: ferrets colonized by *Helicobacter* *mustelae*, which undergo a similar sequence of pathology that leads to gastric adenocarcinoma (Fox et al., 1990, 1997); Mongolian gerbils infected with *H. pylori*, which develop some specific pre-neoplastic lesions, including intestinal metaplasia (Honda et al., 1998b); and Syrian hamsters infected with *Helicobacter aurati* (Patterson et al., 2000).

In veterinary medicine, dogs and cats frequently undergo gastric biopsy to investigate unresolved gastrointestinal disease. In both species, NHPHs are frequently observed, and genetic studies show that these organisms are often present as a mixed infection of different NHPHs (Priestnall et al., 2004) with *H. felis*, *H. heilmannii*, *Helicobacter bizzozeronii* and *Helicobacter salomonis* being the most commonly identified species (Canejo-Teixeira et al., 2014; Priestnall et al., 2004; Van den Bulck et al., 2005).

Although NHPHs, including *H. felis*, are commonly identified in dogs and cats, and are associated with gastritis (Shiratori et al., 2016) and gastric MALT lymphoma in humans (Stolte et al., 2002), genetic studies have shown limited evidence for zoonosis (see Glossary, Box 1). The most frequently isolated *H. heilmannii* from dogs and cats are distinct from the type 1 *H. heilmannii* identified in human MALT lymphoma (Priestnall et al., 2004). However, there is evidence of anthroponosis (see Glossary, Box 1) in the *H. pylori* infection of cats; infection has only been reported in cat colonies that live in proximity to humans (Canejo-Teixeira et al., 2014; Handt et al., 1994).

In dogs, lymphoplasmacytic gastritis is commonly observed in association with NHPHs (Neiger and Simpson, 2000). However, there is no evidence to link conclusively NHPH infection with this gastric pathology. Indeed, NHPH infection is present in 67-86% of clinically healthy dogs, and in 61-100% of animals presenting with chronic vomiting (Amorim et al., 2016). A small-scale study reported that NHPHs are found in association with all cases of canine gastric polyps (Taulescu et al., 2014). Similarly, NHPHs have been found in the stomach of 42-100% of healthy cats, and in 53-76% of those presenting with clinical signs of gastrointestinal disease (Norris et al., 1999). It is hypothesized that these infections play a role in the development of feline gastric lymphoma (Bridgeford et al., 2008).

*Helicobacter suis* is detected in the stomach of up to 80% of pigs at the time of slaughter, with ulceration and hyperkeratosis of the pars oesophagea (see Glossary, Box 1) present in 20-90% of slaughtered pigs (De Bruyne et al., 2012). Although some studies have associated *H. suis* with increased severity of gastritis and with reduced weight gain in pigs (De Bruyne et al., 2012), gastritis is likely to be multifactorial and also involves feed particle size, highly fermentable carbohydrates and stress factors. Interestingly, *H. suis* is the most commonly isolated NHPH found in human stomachs, suggesting the potential for zoonotic transmission.

*Helicobacter*-like DNA has also been isolated from the stomach of thoroughbred horses (Contreras et al., 2007), although the role of *Helicobacter* in gastritis and gastric ulceration in horses is unclear. Horses possess a much larger proportion of non-glandular squamous mucosa than do pigs, which constitutes the proximal half of the stomach mucosa, and gastric ulceration is present in up to 86% of training racehorses (Begg and O'Sullivan, 2003). The high proportion of horses suffering from ulceration that undergo strenuous exercise suggests that stress, management and training practices are likely risk factors (Murray et al., 1996). Ulceration occurs most commonly in the non-glandular portion of the stomach, close to the transition of the non-glandular and glandular stomach, although pyloric ulceration is also observed in 47% of horses (Begg and O'Sullivan, 2003).

Up to 100% of adult ferrets harbour gastric *Helicobacter mustelae*, (Fox et al., 1990); however, this organism is rarely found in ferrets of less than 6 weeks of age (Fox et al., 1988). The incidence of gastric ulceration in ferrets varies between 1.4 and 35% (Andrews et al., 1979), and *H. mustelae* has also been associated with adenocarcinoma (Fox et al., 1997) and gastric lymphoma (Erdman et al., 1997) in this species.

Captive rhesus macaques are commonly infected with *H. pylori*, (Drazek et al., 1994), and non-human primates have been used as models of *H. pylori* infection. Indeed, rhesus macaques in social housing rapidly acquire *H. pylori* from other infected individuals (Solnick et al., 2003). Neonatal rhesus macaques are more commonly infected with *H. pylori* when born to infected mothers, suggesting that close contact in the peripartum period is important for bacterial transmission (Solnick et al., 2003), potentially via an oral-oral route. (Solnick et al., 2006). The induced pathology in rhesus macaques is also very similar to that observed in humans with *H. pylori* infection (Haesebrouck et al., 2009). However, no NHPH species have been uniquely associated with non-human primate gastric colonization, although *H. suis* has been demonstrated in captive mandrills (*Papio sphinx*), cynomolgus monkeys (*Macaca fascicularis*), and in a rhesus macaque (*Macaca mulatta*) from a zoo (Haesebrouck et al., 2009). The question of whether these NHPHs are implicated in the development of gastritis in non-human primates remains unknown. More recently, a study identified a high incidence of gastric adenocarcinoma in a captive colony of sooty mangabeys (*Cercebus atys*) (Sharma et al., 2011). This colony has subsequently been shown to be heavily colonized with *H. suis* by both fluorescence *in situ* hybridization and 16S ribosomal RNA sequencing (Esmail et al., 2016). This is the first evidence of naturally occurring *Helicobacter* associated with gastric carcinogenesis in a non-human primate.

## *In vivo* models of *Helicobacter*-induced gastric pathology

**Fig. 4.**
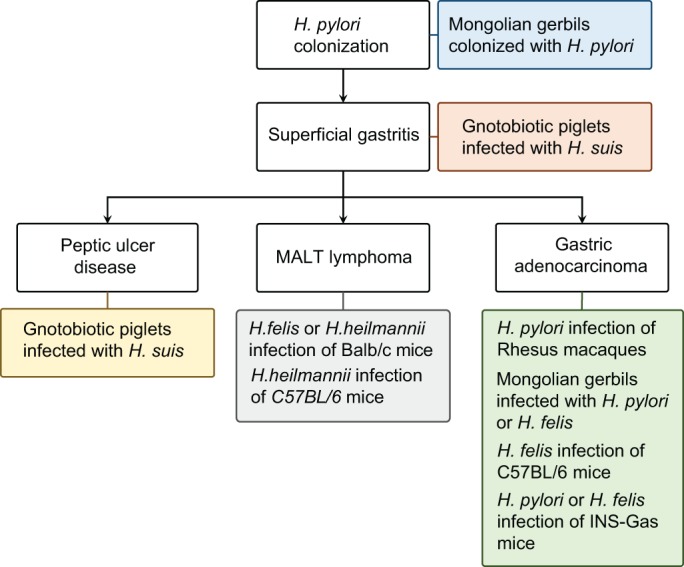
**Modelling the pathological outcomes of *Helicobacter* infection.** A schematic of the principal pathological outcomes of *Helicobacter* infection in humans, annotated with details of the best-characterized *in vivo* models for these conditions.

Because of the breadth of potential pathological outcomes that can follow an *H. pylori* infection, no single animal model can replicate all of the pathological outcomes of this condition. However, as we discuss in more detail below, models do exist that can replicate each of the potential outcomes of *H. pylori* infection in humans (Fig. 4).

### Models of superficial gastritis

The acute phase of *Helicobacter* gastritis has rarely been the focus of research, partly because few cases are reported in the human literature. Where data have been published, they have focused on defining the bacterial and host factors that influence *Helicobacter* colonization and the acute cytokine milieu induced by these bacterial infections.

Historic studies in gnotobiotic pigs demonstrated that the urease enzyme produced by *Helicobacter* bacteria (Eaton and Krakowka, 1994) and their functioning flagellae are essential for effective colonization of the gastric mucosa (Eaton et al., 1992).

Experimental infections of cats with *H. pylori* have shown that active colonization of the gastric mucosa occurs readily, and that a chronic gastritis can follow (Fox et al., 1995). Independently, researchers characterized the acute cytokine response of cats born into a colony of *H. pylori-*infected animals. In this study (Straubinger et al., 2003), the researchers compared animals born into their *H. pylori*-infected colony with specific-pathogen-free (SPF) animals from elsewhere. They demonstrated that animals in the infected colony became colonized with *H. pylori* passively by 14 weeks of age, and that this was associated with an immune response dominated by the expression of cytokines IFN-γ, IL-1α, IL-1β and IL-8. As with the other large animal models of *H. pylori* infection, these experiments benefit from the availability of serial endoscopic evaluation, but are challenging to perform because of the ethical and cost limitations associated with using large animals in experimental procedures.

More recently, Mongolian gerbils have been used to investigate host factors that allow optimal gastric colonization (Bücker et al., 2012). Gastrostomies were performed under terminal anaesthesia to place an intraluminal pH probe, and an auto-titrator into the stomach. The authors used this system to recapitulate the effect on gastric pH of a meal whilst simultaneously inoculating *H. pylori*. Three physiological conditions were replicated using this apparatus. First, it replicated the gastric pH profile observed in newborn human infants, who have a persistently elevated gastric pH due to the large buffering capacity of a milk-based diet and relatively low gastric acid secretion. Second, it replicated the pH profile of young children, in whom gastric pH is transiently elevated as a result of the buffering capacity of a high-milk diet, but then lowered to pH 1-2 by gastric acid secretion. And finally, it replicated the profile of adults, in whom luminal pH remains low throughout the post-prandial period because of high acid secretion and the relatively low buffering capacity of food. The pH profile similar to that of young children enhanced the ability of *H. pylori* to colonize the stomach up to 15-fold, supporting other evidence that the commonest mode of *H. pylori* colonization is through vertical transmission from parent to child (Suerbaum and Josenhans, 2007). This model provides the potential to look at other elements of gastric microbiotal colonization. For instance, does an achlorhydric stomach offer a niche for colonization with other non-*Helicobacter* organisms, and if so, does co-infection with *H. pylori* help or hinder this process?

The immediate host response to colonization with *Helicobacter* has also been modelled by the introduction of *H. pylori*-derived lipopolysaccharide into the stomachs of Sprague-Dawley rats. This induced an inflammatory cell infiltrate typified by lymphocyte infiltration, and increased gastric epithelial cell apoptosis over the course of 4 days. Rather than recapitulating the pathology observed in acute human *H. pylori* infection, the pathological description of this model was more in keeping with the pathology observed in people with chronic superficial gastritis (Slomiany et al., 1998).

These studies have remained relatively niche areas of investigation, and to date have not been replicated by other groups. Significant research questions remain, particularly regarding the initial host responses to *H. pylori* exposure.

### Models of gastric ulceration

*In vivo* models of gastric ulceration induced by *Helicobacter* infection alone are limited to gnotobiotic pigs, Mongolian gerbils and isolated reports of murine gastric ulceration. Several groups have independently shown that gnotobiotic pigs develop ulcers at the junction between the squamous epithelium of the pars oesophagea and columnar mucosa of the true stomach, following infection with *Helicobacter* species harvested from commercially reared pigs (Krakowka et al., 1995, 2005; Kronsteiner et al., 2013). These models have helped to confirm the association between *Helicobacter* colonization and peptic ulceration, but have not been adopted more widely for mechanistic studies.

Mongolian gerbils reportedly develop a wide spectrum of *Helicobacter*-induced pathologies, including gastric ulceration. For example, Honda et al. (1998a) reported that 4 out of 5 gerbils developed gastric ulceration 6 months after colonization with the CagA^+^ (see Glossary, Box 1) *H. pylori* strain, ATCC-43504. Independently, Ogura and colleagues (2000) identified gastric ulceration in 22 out of 23 gerbils infected with the TN2 strain of *H. pylori* for 62 weeks. When the *H. pylori* virulence factor CagE was deleted, none of the 22 infected animals developed gastric ulceration in the same timescale, demonstrating the utility of Mongolian gerbils for modelling gastric ulceration, and the value of this model for characterizing the virulence factors of *H. pylori*.

Most of the literature reporting *H. pylori* colonization of mice suggests that colonization is usually transient, and if persistent, that it is often associated with only mild gastritis (Lachman et al., 1997). In contrast, Kaur et al. (2014) reported a model of gastric ulceration following colonization of female C57BL/6 mice with *H. pylori* DSMZ 10242 for 8 weeks. This study reported multifocal gastric antral ulceration with relatively deep ulcers at 8 weeks, which, if left untreated did not heal. Whilst this study contrasts with much of the literature, it might reflect how factors in an individual laboratory, in particular the baseline microbiota, can influence the outcome of infection in different institutions.

A more established method for investigating the effect of *H. pylori* on gastric ulceration has been to investigate the impact of *H. pylori* infection on chemically induced gastric ulcers. For instance, rats administered acetic acid to the serosal surface of the stomach develop ulcers that heal over several weeks. Gastric colonization with both CagA^+^ (ATCC 43504) (Bui et al., 1991) and CagA^−^ (AH69) (Li et al., 1997) strains of *H. pylori*, impairs the healing of acetic acid-induced ulcers in Sprague-Dawley rats. In this model, extracted *H. pylori* surface proteins (Brzozowski et al., 1999) also impaired the healing of gastric ulcers, suggesting that a response to bacterial components, and not necessarily to the presence of live *Helicobacter*, can impair mucosal healing following gastric injury (Brzozowski et al., 1999).

### Models of gastric adenocarcinoma and its precursor lesions: large and small mammals

Gastric carcinogenesis has been the most extensively studied outcome of *H. pylori* infection, and it has the most diverse array of *in vivo* models, several of which are used in laboratories across the world. Much of the original work investigating the pathogenesis of *Helicobacter*-induced gastric cancer was performed in large animals.

Large animal models of gastric pathology offer the opportunity to perform serial endoscopic biopsies during an experiment. This allows investigators to document the temporal development of gastric pre-neoplasia in individual animals, and has been adopted in beagle dogs (Rossi et al., 1999) and Rhesus monkeys (Liu et al., 2009).

In one such study (Rossi et al., 1999), conventionally housed dogs were infected with a CagA^+^ strain of *H. pylori* (SPM326s) and underwent endoscopic evaluation 1, 2, 4, 8, 12, 18 and 24 weeks after infection. From 8 weeks, chronic superficial gastritis developed, with progressive changes observed in the gastric mucin composition, in keeping with functional gastric atrophy. The authors interpreted this as the development of early pre-neoplastic pathology. The study also demonstrated progression towards atrophic gastritis; however, it is not possible to predict whether more-advanced pre-neoplastic lesions would subsequently develop in this model. This study was also compromised by a lack of detail about the pre-infection gastric microbiota of the animals used in this study; they were demonstrated to be *H. pylori* seronegative, but pre-infection gastric colonization was not assessed for *H. pylori* or for other NHPHs.

*H. pylori* infection of rhesus macaques has been studied in combination with the administration of the ethylating agent, N-ethyl-N′-nitro-N-nitrosoguanidine (ENNG). ENNG is similar to N-nitroso compounds present in traditional Far Eastern diets, and is proposed to be a potential dietary risk factor for developing gastric cancer (Seel et al., 1994). Rhesus macaques were observed over 5 years following *H. pylori* colonization, and gastroscopy was performed quarterly. Neither the continuous administration of ENNG in food *ad libitum*, nor *H. pylori* infection alone induced gastric pre-neoplasia. However, the administration of both agents together induced intestinal-type metaplasia after 2 years of treatment, and more advanced neoplasia, including high-grade dysplasia, in one animal after 5 years. This study demonstrates synergy between ENNG and *H. pylori*, but the study design was unable to determine whether both agents are essential for gastric carcinogenesis, or whether one carcinogen accelerated the effect of the other (Liu et al., 2009).

Although studies using large animal models provided insights into the development of *Helicobacter*-induced gastric cancer, they have not been widely adopted because of the need for specialized animal facilities and the associated high costs. As a result, rodent models are most commonly used. In particular, Mongolian gerbil and murine models have been used frequently in different laboratories to investigate diverse aspects of gastric carcinogenesis.

Both mice and gerbils develop stereotypical, pre-neoplastic pathology in response to *Helicobacter* infection. Whilst the pathways observed in humans and gerbils are similar, there is a difference during the metaplastic phase of gastric pre-neoplasia in mice. In humans and gerbils, the commonest metaplasia is intestinal-type metaplasia. This is characterized by the presence of goblet cells and by the expression of appropriate intestinal markers, such as trefoil factor 3 (TFF3) and mucin 2 (MUC2) (Ectors and Dixon, 1986), as well as by the intestinal differentiation regulating transcription factor CDX2 (Barros et al., 2011; Silberg et al., 2002).

Another metaplastic lesion, defined as spasmolytic polypeptide-expressing metaplasia (SPEM), is less frequently identified in people with gastric pre-neoplasia. It is characterized by a phenotype similar to the secreting Brunner's glands of the intestine, or to deep antral gland cells that express MUC6 and trefoil factor 2 (TFF2, or spasmolytic polypeptide) (Weis and Goldenring, 2009). In gerbils, the distribution of these lesions is similar to that observed in humans (Yoshizawa et al., 2007), whereas C57BL/6 mice infected with *H. felis* develop a predominantly SPEM response, with little or no evidence of intestinal-type metaplasia (Shimizu et al., 2016; Weis and Goldenring, 2009).

When colonized by *H. pylori* and by several other NHPH strains, including *H. felis*, *H. bizzozeroni* and *H. suis* (De Bock et al., 2006a,b; Liang et al., 2015; Nakamura et al., 2007), Mongolian gerbils reportedly develop advanced pre-malignant lesions. However, the use of this organism is complicated by problems of experimental reproducibility. For example, Watanabe and colleagues (1998) reported that 5 out of 5 gerbils infected with the CagA^+^ *H. pylori* strain TN2GF4 in a standard animal house environment for 52 weeks developed intestinal metaplasia, and 10 of 27 gerbils infected for 62 weeks developed invasive adenocarcinomas. In contrast, Elfvin et al. (2005) studied gerbils that were colonized with either *H. pylori* SS1 or TN2GF4 and maintained in a SPF facility for up to 18 months. None of these animals developed invasive adenocarcinoma, and only 2 of 5 infected animals at 12 months, and 3 of 10 infected animals at 18 months, developed intestinal metaplasia.

Because the Mongolian gerbil is outbred, the genetic backgrounds of animals supplied to different laboratories up to a decade apart, will have been divergent. The conditions within different animal units might also have contributed to differences in these studies, particularly differences in diet and resident microbiota, which could influence host pathology. These observations demonstrate some of the challenges of comparing *in vivo* studies performed in different environments, and challenge the received wisdom that experimental results should always be reproducible in different settings (discussed further in Justice and Dhillon, 2016; Schofield et al., 2016). Fully understanding why these apparently similar experiments resulted in divergent outcomes could offer insights into the mechanisms that drive the development of gastric pre-neoplasia in the gerbil.

The Mongolian gerbil has been particularly useful for identifying *Helicobacter*-specific and environmental factors that influence the development of gastric cancer. It has been used to demonstrate the carcinogenic potential of both *H. pylori* (Watanabe et al., 1998) and *H. felis* (Court et al., 2002; De Bock et al., 2006a,b), and that *H. bizzozeronii* and *H. salomonis* (De Bock et al., 2006b) are less likely to induce gastric pre-neoplasia in this species. In addition, several studies have identified that high-salt diets promote the development of *H. pylori*-induced gastric pre-neoplastic pathology in gerbils (Bergin et al., 2003; Kato et al., 2006; Nozaki et al., 2002), consistent with human epidemiological studies (Wang et al., 2009).

The Mongolian gerbil has also been used to adapt *H. pylori* strains to a rodent environment. This has been demonstrated best by the passage of *H. pylori* B128, derived from a patient with gastric ulceration, through a male gerbil for 3 weeks. *H. pylori* was subsequently re-cultured from this animal's stomach and described as *H. pylori* strain 7.13, which is more pathogenic than strain B128. Six of eight gerbils infected with *H. pylori* 7.13 developed gastric adenocarcinoma 8 weeks after inoculation, in comparison to none in the B128 group (Franco et al., 2005). The same researchers demonstrated that this phenotype was essentially reproducible, and preventable by eradication of *H. pylori* (Romero-Gallo et al., 2008). The 7.13 strain has been further characterized by genome sequencing (Asim et al., 2015), providing data that will help to advance our future understanding of *H. pylori* pathogenicity.

The laboratory mouse is the other rodent used extensively to model gastric pre-neoplasia. *H. pylori* colonization of C57BL/6 inbred mice leads to gastritis with epithelial cell hyperplasia, but this does not progress to dysplasia or to cancer (Lee et al., 1997). However, the colonization of C57BL/6 mice with *H. felis* is consistently shown to lead to gastric pre-neoplasia, and when infections have persisted for 13-15 months, adenocarcinomas have been reported (Fox et al., 2002). This outcome is specific to the C57BL/6 genetic background.

Other strains of inbred mouse, including Balb/c, respond differently to *H. felis* infection*.* This strain can be colonized by *H. felis*, but does not develop gastric pre-neoplastic pathology (Wang et al., 1998). The mechanisms underlying these differences between mouse strains are attributable to differences in immune response. C57BL/6 mice demonstrate a Th1-polarized immune response, whilst Balb/c mice have a more Th2-polarized response to *H. felis* colonization, which allows infection to persist but does not promote chronic epithelial disruption (Wang et al., 1998). The B6129 mouse, generated by crossing C57BL/6 with 129S6/SvEv, is particularly sensitive to *Helicobacter*-induced pathology triggered by *H. felis*. In these mice, gastric intraepithelial neoplasia developed 8 months after *H. pylori* infection whereas malignant tumours developed after ∼15 months (Rogers et al., 2005).

In addition to the models of gastric cancer induced solely by *Helicobacter* infection, several chemically induced gastric cancer models are accelerated by co-infection with *H. pylori*. These include mice and Mongolian gerbils infected with *H. pylori* and treated with N-methyl-N-nitrosourea, (MNU), and Mongolian gerbils infected with *H. pylori* and treated with the carcinogen methylnitronitrosoguanidine (MNNG). In Mongolian gerbils, the co-administration of *H. pylori* with MNNG accelerated the carcinogenic process, leading to invasive cancer in 60-80% of animals treated for 1 year (Maruta et al., 2001, 2000; Tatematsu et al., 1998). In mice, the effect of co-administering MNU and *H. pylori* is less clear, with contrasting reports of either synergy between the two stimuli (Han et al., 2002) or no additional effect above chemical carcinogen alone (Nakamura et al., 2002).

### Transgenic mouse models of gastric carcinogenesis

Several transgenesis strategies have been used to study gastric carcinogenesis. These include the induction of spontaneous gastric atrophy, the expression of *H. pylori* pathogenicity factors, and the overexpression of known oncogenes in the gastric mucosa. Transgenic animals have also been used to explore the role of specific molecular pathways that potentially modulate gastric carcinogenesis. A complete description of the genetically engineered mouse models (GEMMs) used in gastric carcinogenesis research is beyond the scope of this article, and so we refer readers to another recent review for more information (Jiang and Yu, 2017). Here, we focus on the best characterized of these models.

Amongst the most established examples of transgenically induced gastric atrophy is the INS-Gas mouse model [also known as FVB-tg(rl1-hinsgas) (Wang and Brand, 1992)]. This mouse expresses a human gastrin mini-gene under the control of the rat insulin promoter. It constitutively expresses gastrin in β-cells of the pancreatic islets, resulting in the constitutive over-production of amidated gastrin. These animals are born with increased numbers of parietal cells and at birth hypersecrete acid, but over the first 5 months of life, parietal cell numbers fall to that of wild-type animals. Over longer periods, profound gastric atrophy occurs, and a proportion of these animals develop spontaneous gastric cancers (Wang et al., 2000). *Helicobacter* infection accelerates the gastric pathology observed in this mouse; 85% of INS-Gas mice infected with *H. felis* developed gastric cancers 7 months after infection (Wang et al., 2000). Similarly, when INS-Gas mice are infected with *H. pylori*, they exhibit significantly more severe gastric inflammation and dysplasia relative to uninfected controls. Interestingly, INS-Gas males are more severely affected by *H. pylori* infection than are female mice, for reasons that are unknown, and in the same study, mice exposed to high dietary salt levels had more-severe gastric pathology relative to untreated controls (Fox et al., 2003).

More recently, the INS-Gas mouse has been used to characterize the role that the non-*Helicobacter* microbiome has during gastric carcinogenesis. INS-Gas mice bred and maintained in a germ-free environment did not develop spontaneous gastric pre-neoplasia, while otherwise germ-free mice infected with *H. pylori* developed fewer tumours than did *H. pylori*-infected SPF mice (Lofgren et al., 2011). Subsequently, the same group demonstrated that a limited group of three bacterial species is sufficient to restore the phenotype of SPF mice in otherwise germ-free mice (Lertpiriyapong et al., 2014), and that co-infection with the intestinal roundworm *Heligmosomoides polygyrus* can protect INS-Gas mice against *H. pylori*-induced gastric pre-neoplasia (Whary et al., 2014). This protection was associated with an increase in the number of cells positive for forkhead box P3 (FOXP3) – a master regulator of regulatory T-cell (T_reg_) development – in the gastric corpus of mice, suggesting a possible shift in the T_reg_ immune response to *H. pylori* infection.

The CEA/SV40T L5496 mouse expresses the SV40T proto-oncogene under the control of a truncated carcinoembryonic antigen (CEA) promoter. Gastric tumours formed in all mice, with dysplasia evident as early as 37 days postnatally and with animals becoming moribund due to gastric tumour burden by 100-130 days postnatally (Thompson et al., 2000).

Mice transgenically deficient for *mutT* homolog-1 (*Mth1*) are unable to process oxygen free radicals, which cause DNA damage, and are susceptible to several spontaneous tumours, including gastric tumours, which develop over ∼18 months; 14% (13 of 93) of *Mth1*-null mice developed gastric tumours, compared with 4% (4 of 90) of littermate controls (Tsuzuki et al., 2001).

In Trefoil factor 1 (*Tff1*)-deficient mice, the structure of both the antral and corpus mucosa is markedly altered from birth. By 5 months of age, all *Tff1*-null mice exhibit adenomatous changes in the gastric mucosa, and 30% have established adenocarcinomas (Lefebvre et al., 1996).

The C57BL/6J-tg(H/K_ATPase/hIL-1β) mouse overexpresses human interleukin 1B (*IL1B*) under the control of the H^+^/K^+^ ATPase β-subunit promoter, leading to expression of human IL1-β exclusively in parietal cells. In this model, 15% of male mice develop spontaneous tumours at 14 months of age, and disease severity is markedly increased by infection with *H. felis* (Tu et al., 2008).

Transgenic mutation of the IL6 co-receptor, *Gp130*, leads to a severe gastric phenotype in which gastric antral adenomas spontaneously develop over the first 6-8 weeks of life, and subsequently grow and spread to include the gastric fundus by 48 weeks of age (Judd et al., 2004; Tebbutt et al., 2002). Whilst this animal does not exhibit the classical pre-neoplastic pathology induced by *Helicobacter* infection, it may have relevance to *H. pylori-*mediated disease because CagA status has been shown to influence gp130-mediated switching between the MAPK/ERK (stimulated by the tyrosine phosphatase SHP2) and JAK-STAT signalling cascades *in vitro* (Lee et al., 2010). Signalling through these mechanisms influences a number of cellular processes that are altered in cancer, including regulation of cell proliferation and apoptosis, invasion and angiogenesis. Disruption of these pathways, either by direct mutagenesis or through other mechanisms, has been identified in many different tumour types (Zhang et al., 2015).

Transgenic expression of the *H. pylori* virulence factor CagA is oncogenic to the gastric mucosa, both when expressed constitutively throughout the animal under the control of the β-actin promoter [C57BL/6-tg(CAG-CagA^Hs^)], and when limited to the gastric mucosa under the control of the H^+^/K^+^ ATPase β-subunit promoter [C57BL/6J-tg(HK-CagA^Hs^)]. In both cases, animals developed gastric hypertrophy by 12 weeks of age, and over 3 years, developed gastric dysplasia or occasionally gastric adenocarcinomas (Ohnishi et al., 2008).

### Models of MALT lymphoma

Several factors make *Helicobacter*-induced MALT lymphoma challenging to model. It is a rare outcome of *H. pylori* infection; it develops after a prolonged, complex interaction among the bacteria, host epithelium and host immune system; and the commonest, relatively indolent form of MALT lymphoma is challenging to diagnose pathologically. These issues are compounded by the fact that natural *Helicobacter*-induced MALT lymphomas have not been reported in commonly used laboratory species. Consequently, in some cases, only one research group has assessed the models described below, and substantial gaps remain in our knowledge of the mechanisms involved in *H. pylori*-induced gastric MALT lymphoma formation.

Within these studies, there is also heterogeneity in the criteria used to report MALT lymphoma formation. Most studies describe lymphoepithelial lesions as a pathognomic event, signifying the initiation of lymphomagenesis. Other studies used evidence of monoclonal lymphoid aggregate formation as a surrogate for the development of MALT lymphoma.

Experimental induction of MALT lymphoma by *H. pylori* was reported in a single study of conventionally housed beagle dogs. This study described the formation of monoclonal lymphoid aggregates in the gastric mucosa of dogs infected with *H. pylori* strain SPM326s (CagA^+^) for 6 months. This study was not extended to later time points, and in the absence of epithelial destruction or evidence of genetic instability, the association with MALT lymphoma remains somewhat tenuous (Rossi et al., 1999). In addition, the pre-existing gastric *Helicobacter* status of the dogs at the study onset was not evaluated, although seropositivity for *H. pylori* was excluded. It is therefore not possible to conclude whether the observed phenotype was due to *H. pylori* infection in isolation or to a synergy between *H. pylori* and other gastric *Helicobacter* species.

Several NHPHs have been reported to induce either MALT lymphoma or precursor lesions in laboratory conditions. Two of six Mongolian gerbils infected with the *H. suis* strain, HS5, for 9 months developed lymphoepithelial lesions (Flahou et al., 2010), whilst outbred Swiss, and inbred Balb/c and C57BL/6, mice infected with a variety of NHPH strains developed lymphoepithelial lesions.

In one of these studies, Enno et al. (1995) reported that 25% of Balb/c mice colonized with *H. felis* for 22-26 months developed advanced lymphoepithelial lesions, and a further 5% had early lymphoepithelial lesions. These findings have since been independently replicated, and in the same study, a variety of *H. heilmannii* strains isolated from various sources, including mandrill monkeys and bobcats, also induced gastric MALT lymphomas over a similar timescale (O'Rourke et al., 2004).

A commonly used model of gastric carcinogenesis is the long-term infection of C57BL/6 mice with *H. felis*; however, these mice have not been reported to develop gastric MALT lymphoma. They do, nevertheless, develop gastric lymphoepithelial lesions and low-grade MALT lymphomas when colonized for 1 year with a candidatus *H. heilmannii* isolated from the stomach of a cynomolgus monkey (Nakamura et al., 2007). More recently, we have demonstrated that 50% (3/6) of C57BL/6 mice lacking the c-Rel NF-κB subunit developed early lymphoepithelial lesions when colonized with *H. felis* for 12 months (Burkitt et al., 2013). This pathology has not previously been reported in the C57BL/6/*H. felis* model, suggesting that signalling through the c-Rel NF-κB subunit could influence the regulation of gastric MALT lymphoma formation.

Overall, these data support the hypothesis that NHPHs play a specific role in the development of MALT lymphoma, and since several of the typical *H. pylori* virulence factors are not expressed in these *Helicobacter* species, this suggests that novel bacterial factors might be important in the development of gastric MALT lymphoma. Future work in this field needs to incorporate models of genetic instability, in addition to morphological criteria, to strengthen the quality of data generated from these models.

## *Ex vivo* models of *Helicobacter* infection of the stomach

To date, most studies investigating the mechanisms that underlie *H. pylori*-induced gastric pathology have relied on *in vivo* models. Many of these models require prolonged exposure to *Helicobacter* and use relatively large numbers of animals, raising questions of animal welfare and cost. In addition, studying the interaction of two whole organisms (and increasingly the rest of the microbiome to which *H. pylori* contributes) generates hugely complex systems. Some studies have tried to address this complexity by using elegant transgenic mouse systems, for example, by using tissue-specific transgenesis. However, these systems remain highly complex, and genetic manipulation can introduce further complexity, either through gene expression in a suboptimal location, or through off-target effects of the drugs used to induce genetic recombination.

There is, therefore, a need for better *in vitro* or *ex vivo* models of *H. pylori-*associated pathology. Over the past 6 years, the development of first murine, and subsequently human, three-dimensional, primary gastric gland cultures called gastric organoids or ‘gastroids’ (see Glossary, Box 1) has opened up the prospect of using untransformed, gastric tissue in culture to model the development of gastric pathology.

### *Ex vivo* gastric mucosal culture models

In order to generate gastric glandular units in culture, it must first be possible to isolate primary material from an organism, to passage this material *in vitro*, and then demonstrate its ability to differentiate into the different cell types of the gastric epithelium. Ideally, it should also be possible to store the cultures in the laboratory and reconstitute them, deriving reproducible results from cultures that have been stored or not stored.

Over the past decade, culture systems have been developed that fulfil these criteria. The key discovery came with the identification of LGR5 as a marker of gastrointestinal stem cells (Barker et al., 2010, 2007). The identification of this WNT-signalling family member as a key marker of gastrointestinal stem cells led rapidly to the development of primary culture systems that support these cells primarily through the optimization of WNT signalling. Since these discoveries, several groups have established gastric organoid systems using slightly different approaches (McCracken et al., 2014; VanDussen et al., 2015), as described below.

### Long-lived gastric epithelial cultures derived from primary gastric tissue

The generation of self-renewing gastric gland cultures was first described by Barker, using methodology developed from earlier intestinal organoid models (see Glossary, Box 1) established by the same group. This method uses LGR5^+^ stem cells extracted from the gastric antrum of mice expressing green fluorescent protein (GFP) under the control of the *Lgr5* promoter as starting material. These cells are supported *in vitro* in a 3D matrix together with recombinant growth factors that together recapitulate the stem cell niche. In addition to activating the WNT pathway, TGFβ pathway signalling is suppressed and gastrin 17 (GAST) and fibroblast growth factor 10 (FGF10) are added as gastroid-specific growth factors. During the initial phase of culture, the ROCK inhibitor Y-27632 is added to the growth medium to prevent anoikis. This methodology established LGR5^+^ cells as gastric antral stem cells; intriguingly, an effective marker for the gastric corpus stem cell remains elusive.

**Fig. 5.**
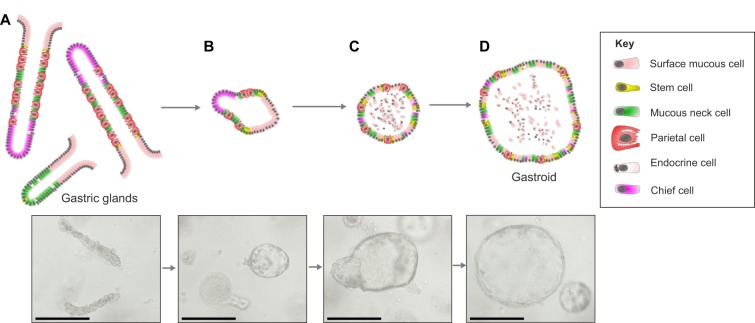
**Gastric organoid culture and differentiation.** Diagrams and images showing the maturation of gastric organoids. (A) Freshly digested gastric corpus glands from a C57BL/6 mouse. (B) Glands 24 h after harvesting that have formed immature organoids and have a small spherical appearance. (C) On day 3 of culture, the immature organoids have expanded and can be passaged. (D) Following passage, the organoids retain their spherical appearance and continue to grow. Images from authors' own laboratories. Scale bars: 250 µm.

Subsequent protocols have established similar methods for the establishment of murine gastric organoid cultures from both antrum and corpus using non-enzymatically dissociated gastric glands as starting material (Mahe et al., 2013), and for organoids based on gastric tissue samples taken at the time of gastric resection (Bartfeld and Clevers, 2015; Schlaermann et al., 2016). These cultures generate spherical cultures that maintain a 3D structure in culture (Fig. 5).

A modification of this protocol uses conditioned medium from the L-WRN cell line, which secretes human WNT3A, noggin (NOG) and r-spondin 3 (RSPO1) (VanDussen et al., 2015). This system has been shown to allow cultures to be established from small samples taken during an endoscopic examination of the stomach, rather than requiring samples to be excised during surgery, and could be more cost effective because of the high cost of recombinant growth factors. However, the use of this cell line limits the control that an experimentalist can have on the culture system; in particular, gastric organoids grown using this method are exposed to particularly high levels of WNT3A, making the cultures more proliferative and less likely to differentiate than gastric organoids established using recombinant growth factors.

Most reports of gastrointestinal organoid culture systems to date have retained the 3D structure. However, an increasing number of studies use 3D organoids as the source material to generate epithelial monolayers on collagen-coated glass or plastics. This technique offers different opportunities for quantification and observation of morphology, which in some cases might be easier to relate back to more-established 2D cancer cell cultures (Schlaermann et al., 2016).

### Long-lived gastric epithelial cultures derived from induced pluripotent stem cells

Human gastric organoids have recently been generated from both human embryonic stem cell lines and induced pluripotent stem cell (iPSC) lines (McCracken et al., 2014). These stem cells were first differentiated into definitive endoderm before the induction of the foregut marker SOX2, by exposure to WNT3A, FGF4 and noggin. They were further differentiated into ‘antral’- and ‘corpus’-type cultures by exposure to retinoic acid and subsequently differentiated into mature organoids using epidermal growth factor (EGF).

Both antral- and corpus-type gastric organoids generated from such cultures are similar to their originating tissues, as shown by microarray and by gene set enrichment analyses (McCracken et al., 2014). Morphologically, antral gland type organoids also contain identifiable epithelial and endocrine cell types. Corpus-type organoids do not possess parietal cells; however, other markers of gastric corpus tissue, including expression of pepsinogens and ghrelin, are detectable.

### The effect of *Helicobacter* infection on cultured gastric organoids

Several groups have investigated the impact of *H. pylori* infection on gastric organoid cultures. The most reproduced finding is that epithelial cell proliferation is enhanced by direct mucosal interaction with *H. pylori*. This has been demonstrated by the microinjection of *H. pylori* into 3D fundic gland organoids derived from both mice and humans (Bartfeld et al., 2015; Bertaux-Skeirik et al., 2015), and into human pluripotent stem cell-derived gastric organoids (McCracken et al., 2014).

*H. pylori* infection also induces morphological changes in 2D gastric-organoid-derived monolayers grown on collagen-coated glass or plastics, with epithelial cells taking on a hummingbird morphology (Schlaermann et al., 2016). This response was CagA dependent and appears to be analogous to the SHP2-mediated hummingbird morphology previously described in gastric cancer cell lines (Higashi et al., 2002). This change in morphology was associated with the activation of the classical NF-κB signalling pathway; this pathway is also implicated in the response of 3D gastric organoids to *H. pylori* microinjection (Schlaermann et al., 2016; Schumacher et al., 2015). In gastric cancer cell lines, this morphology is associated with a more aggressive, invasive phenotype (Chang et al., 2016) and epithelial-mesenchymal transition (Snider et al., 2008).

Sigal et al. (2015) have established an organoid-formation assay in which they quantified the percentage of viable organoids formed from a preparation. Using this assay, they verified their own observation that the antral LGR5^+^ stem cell zone expands in response to *H. pylori* infection. This provides a novel method for quantifying the abundance of antral stem cells.

A further study investigating the interactions between *H. pylori* and human gastric cells in the context of organoids has demonstrated that *H. pylori* can sense nanomolar concentrations of urea, and use this as a chemoattractant. This study made use of the observation that whilst most organoids form with the luminal surface of the epithelium facing inwards, a small proportion form with an ‘inside-out’ structure. This allowed the authors to observe epithelial and bacterial interactions, and in particular the adherence of *H. pylori* to cell-cell junctions (Huang et al., 2015).

## Conclusions

An ever more diverse array of laboratory models exists to explore *H. pylori*-induced pathology. The course of infection and plethora of outcomes in current *in vivo* models are probably too complex to understand fully using current technology. The new generation of *ex vivo* models offer opportunities for researchers to be more systematic in their approach; however, at present the models also risk being reductionist. Over time we need to develop *ex vivo* systems that can be interrogated systematically, but which incorporate key elements of *in vivo* models, including host epithelial, mesenchymal and immune compartments, and both *Helicobacter* and non-*Helicobacter* microbiota.

Currently available gastric organoid models have focused largely on the development of organoids from healthy animals and humans, which have then been infected with *H. pylori*. However, future studies will need to develop models that mimic the development of other gastric epithelial pathologies in culture. In particular, the development of 3D models of gastric atrophy and metaplasia will allow researchers to perform experiments to compare the effect of developing gastric epithelial pathology *in vivo* and *ex vivo*. Being able to make these comparisons will allow better mechanistic studies to be performed in the relatively simple organoid systems and verified in whole animals, avoiding the pitfalls of a reductionist scientific approach.

The results of these studies will begin to provide better data that segregate epithelial events from immune and mesenchymal driven changes in the stomach. Developing these models presents a major challenge for the future but, if successful, they are likely to permit the design and evaluation of new therapeutic strategies for patients who currently have no meaningful treatment options.

## References

[DMM027649C1] Agha A. and Graham D. Y. (2005). Evidence-based examination of the African enigma in relation to *Helicobacter pylori* infection. *Scand. J. Gastroenterol.* 40, 523-529. 10.1080/00365520510012280 16036504

[DMM027649C2] Amorim I., Taulescu M. A., Day M. J., Catoi C., Reis C. A., Carneiro F. and Gärtner F. (2016). Canine gastric pathology: a review. *J. Comp. Pathol.* 154, 9-37. 10.1016/j.jcpa.2015.10.181 26774560

[DMM027649C3] Andrews P. L., Illman O. and Mellersh A. (1979). Some observations of anatomical abnormalities and disease states in a population of 350 ferrets (Mustela furo L.). *Z. Versuchstierkd.* 21, 346-353. 549381

[DMM027649C4] Asenjo L. M. and Gisbert J. P. (2007). [Prevalence of *Helicobacter pylori* infection in gastric MALT lymphoma: a systematic review]. *Rev. Esp. Enferm. Dig.* 99, 398-404. 10.4321/S1130-01082007000700006 17973584

[DMM027649C5] Asfeldt A. M., Straume B., Steigen S. E., Løchen M.-L., Florholmen J., Bernersen B., Johnsen R. and Paulssen E. J. (2008). Changes in the prevalence of dyspepsia and *Helicobacter pylori* infection after 17 years: the Sørreisa gastrointestinal disorder study. *Eur. J. Epidemiol.* 23, 625-633. 10.1007/s10654-008-9275-x 18704703

[DMM027649C6] Asim M., Chikara S. K., Ghosh A., Vudathala S., Romero-Gallo J., Krishna U. S., Wilson K. T., Israel D. A., Peek R. M. Jr and Chaturvedi R. (2015). Draft genome sequence of gerbil-adapted carcinogenic *Helicobacter pylori* strain 7.13. *Genome Announc.* 3, e00641-15 10.1128/genomeA.00641-15 PMC4463538 26067974

[DMM027649C7] Baele M., Decostere A., Vandamme P., Van den Bulck K., Gruntar I., Mehle J., Mast J., Ducatelle R. and Haesebrouck F. (2008). Helicobacter baculiformis sp. nov., isolated from feline stomach mucosa. *Int. J. Syst. Evol. Microbiol.* 58, 357-364. 10.1099/ijs.0.65152-0 18218931

[DMM027649C8] Barker N., van Es J. H., Kuipers J., Kujala P., van den Born M., Cozijnsen M., Haegebarth A., Korving J., Begthel H., Peters P. J. et al. (2007). Identification of stem cells in small intestine and colon by marker gene Lgr5. *Nature* 449, 1003-1007. 10.1038/nature06196 17934449

[DMM027649C9] Barker N., Huch M., Kujala P., van de Wetering M., Snippert H. J., van Es J. H., Sato T., Stange D. E., Begthel H., van den Born M. et al. (2010). Lgr5+ve stem cells drive self-renewal in the stomach and build long-lived gastric units in vitro. *Cell Stem Cell* 6, 25-36. 10.1016/j.stem.2009.11.013 20085740

[DMM027649C10] Barros R., da Costa L. T., Pinto-de-Sousa J., Duluc I., Freund J.-N., David L. and Almeida R. (2011). CDX2 autoregulation in human intestinal metaplasia of the stomach: impact on the stability of the phenotype. *Gut* 60, 290-298. 10.1136/gut.2010.222323 21148572 PMC3034084

[DMM027649C11] Bartfeld S. and Clevers H. (2015). Organoids as Model for infectious diseases: culture of human and murine stomach organoids and microinjection of *Helicobacter pylori*. *J. Vis. Exp.* 105, e53359 10.3791/53359 PMC4692704 26650279

[DMM027649C12] Bartfeld S., Bayram T., van de Wetering M., Huch M., Begthel H., Kujala P., Vries R., Peters P. J. and Clevers H. (2015). In vitro expansion of human gastric epithelial stem cells and their responses to bacterial infection. *Gastroenterology* 148, 126-136.e6. 10.1053/j.gastro.2014.09.042 25307862 PMC4274199

[DMM027649C13] Begg L. M. and O'Sullivan C. B. (2003). The prevalence and distribution of gastric ulceration in 345 racehorses. *Aust. Vet. J.* 81, 199-201. 10.1111/j.1751-0813.2003.tb11469.x 15080440

[DMM027649C14] Ben Mansour K., Fendri C., Battikh H., Garnier M., Zribi M., Jlizi A. and Burucoa C. (2016). Multiple and mixed *Helicobacter pylori* infections: Comparison of two epidemiological situations in Tunisia and France. *Infect. Genet. Evol.* 37, 43-48. 10.1016/j.meegid.2015.10.028 26518911

[DMM027649C15] Bergin I. L., Sheppard B. J. and Fox J. G. (2003). *Helicobacter pylori* infection and high dietary salt independently induce atrophic gastritis and intestinal metaplasia in commercially available outbred Mongolian gerbils. *Dig. Dis. Sci.* 48, 475-485. 10.1023/A:1022524313355 12757158

[DMM027649C16] Bertaux-Skeirik N., Feng R., Schumacher M. A., Li J., Mahe M. M., Engevik A. C., Javier J. E., Peek R. M. Jr, Ottemann K., Orian-Rousseau V. et al. (2015). CD44 plays a functional role in *Helicobacter pylori*-induced epithelial cell proliferation. *PLoS Pathog.* 11, e1004663 10.1371/journal.ppat.1004663 25658601 PMC4450086

[DMM027649C17] Bridgeford E. C., Marini R. P., Feng Y., Parry N. M. A., Rickman B. and Fox J. G. (2008). Gastric Helicobacter species as a cause of feline gastric lymphoma: a viable hypothesis. *Vet. Immunol. Immunopathol.* 123, 106-113. 10.1016/j.vetimm.2008.01.016 18387674 PMC2653416

[DMM027649C18] Brown C. C., Dale C. B. and Barker I. K. (2007). Alimentary system. In *Pathology of Domestic Animals*, Vol. 2 (ed. Maxie M. G.), p. 60 Edinburgh; New York: Elsevier Saunders.

[DMM027649C19] Brzozowski T., Konturek P. C., Konturek S. J., Kwiecien S., Pajdo R., Karczewska E., Stachura J. and Hahn E. G. (1999). Water extracts of *Helicobacter pylori* delay healing of chronic gastric ulcers in rats: role of cytokines and gastrin-somatostatin link. *Digestion* 60, 22-33. 10.1159/000007585 9892795

[DMM027649C20] Bücker R., Azevedo-Vethacke M., Groll C., Garten D., Josenhans C., Suerbaum S. and Schreiber S. (2012). *Helicobacter pylori* colonization critically depends on postprandial gastric conditions. *Sci. Rep.* 2, 994 10.1038/srep00994 23251780 PMC3524519

[DMM027649C21] Bui H. X., del Rosario A., Sonbati H., Lee C. Y., George M. and Ross J. S. (1991). *Helicobacter pylori* affects the quality of experimental gastric ulcer healing in a new animal model. *Exp. Mol. Pathol.* 55, 261-268. 10.1016/0014-4800(91)90006-J 1748215

[DMM027649C22] Burkitt M. D., Varro A. and Pritchard D. M. (2009). Importance of gastrin in the pathogenesis and treatment of gastric tumors. *World J.Gastroenterol.* 15, 1-16. 10.3748/wjg.15.1 19115463 PMC2653300

[DMM027649C23] Burkitt M. D., Williams J. M., Duckworth C. A., O'Hara A., Hanedi A., Varro A., Caamaño J. H. and Pritchard D. M. (2013). Signaling mediated by the NF-kappaB sub-units NF-kappaB1, NF-kappaB2 and c-Rel differentially regulate Helicobacter felis-induced gastric carcinogenesis in C57BL/6 mice. *Oncogene* 32, 5563-5573. 10.1038/onc.2013.334 23975431 PMC3898319

[DMM027649C24] Campbell D. I., Warren B. F., Thomas J. E., Figura N., Telford J. L. and Sullivan P. B. (2001). The African enigma: low prevalence of gastric atrophy, high prevalence of chronic inflammation in West African adults and children. *Helicobacter* 6, 263-267. 10.1046/j.1083-4389.2001.00047.x 11843957

[DMM027649C25] Canejo-Teixeira R., Oliveira M., Pissarra H., Niza M. M. R. E. and Vilela C. L. (2014). A mixed population of *Helicobacter pylori*, *Helicobacter bizzozeronii* and “*Helicobacter heilmannii*” in the gastric mucosa of a domestic cat. *Irish Vet. J.* 67, 25 10.1186/2046-0481-67-25 PMC4405862 25905013

[DMM027649C26] Cattoli G., Bart A., Klaver P. S., Robijn R. J., Beumer H. J., van Vugt R., Pot R. G. J., Vandenbroucke-Grauls C. M. J. E., Kuipers E. J., Kusters J. G. et al. (2000). *Helicobacter acinonychis* eradication leading to the resolution of gastric lesions in tigers. *Vet. Rec.* 147, 164-165. 10.1136/vr.147.6.164 10975334

[DMM027649C27] Chang C.-C., Kuo W.-S., Chen Y.-C., Perng C.-L., Lin H.-J. and Ou Y.-H. (2016). Fragmentation of CagA Reduces Hummingbird Phenotype Induction by *Helicobactor pylori*. *PLoS ONE* 11, e0150061 10.1371/journal.pone.0150061 26934189 PMC4775065

[DMM027649C28] Contreras M., Morales A., García-Amado M. A., De Vera M., Bermúdez V. and Gueneau P. (2007). Detection of Helicobacter-like DNA in the gastric mucosa of Thoroughbred horses. *Lett. Appl. Microbiol.* 45, 553-557. 10.1111/j.1472-765X.2007.02227.x 17908231

[DMM027649C29] Correa P., Haenszel W., Cuello C., Tannenbaum S. and Archer M. (1975). A model for gastric cancer epidemiology. *Lancet* 306, 58-60. 10.1016/S0140-6736(75)90498-5 49653

[DMM027649C30] Court M., Robinson P. A., Dixon M. F. and Crabtree J. E. (2002). Gastric Helicobacter species infection in murine and gerbil models: comparative analysis of effects of *H. pylori* and *H. felis* on gastric epithelial cell proliferation. *J. Infect. Dis.* 186, 1348-1352. 10.1086/344321 12402207

[DMM027649C31] Danesh P. (1999). *Helicobacter pylori* infection and gastric cancer: systematic review of the epidemiological studies. *Aliment. Pharmacol. Ther.* 13, 851-856. 10.1046/j.1365-2036.1999.00546.x 10383517

[DMM027649C32] De Bock M., D'Herde K., Duchateau L., Hellemans A., Decostere A., Haesebrouck F. and Ducatelle R. (2006a). The effect of *Helicobacter felis* and *Helicobacter bizzozeronii* on the gastric mucosa in Mongolian gerbils: a sequential pathological study. *J. Comp. Pathol.* 135, 226-236. 10.1016/j.jcpa.2006.08.003 17069831

[DMM027649C33] De Bock M., Decostere A., Hellemans A., Haesebrouck F. and Ducatelle R. (2006b). *Helicobacter felis* and *Helicobacter bizzozeronii* induce gastric parietal cell loss in Mongolian gerbils. *Microbes Infect.* 8, 503-510. 10.1016/j.micinf.2005.08.003 16311055

[DMM027649C34] De Bruyne E., Flahou B., Chiers K., Meyns T., Kumar S., Vermoote M., Pasmans F., Millet S., Dewulf J., Haesebrouck F. et al. (2012). An experimental *Helicobacter suis* infection causes gastritis and reduced daily weight gain in pigs. *Vet. Microbiol.* 160, 449-454. 10.1016/j.vetmic.2012.06.031 22776514

[DMM027649C35] De Groote D., van Doorn L. J., Ducatelle R., Verschuuren A., Tilmant K., Quint W. G. V., Haesebrouck F. and Vandamme P. (1999). Phylogenetic characterization of ‘Candidatus *Helicobacter bovis*’, a new gastric helicobacter in cattle. *Int. J. Syst. Evol. Microbiol.* 49, 1707-1715. 10.1099/00207713-49-4-1707 10555352

[DMM027649C36] D'Elios M. M., Manghetti M., Almerigogna F., Amedei A., Costa F., Burroni D., Baldari C. T., Romagnani S., Telford J. L. and Del Prete G. (1997). Different cytokine profile and antigen-specificity repertoire in *Helicobacter pylori*-specific T cell clones from the antrum of chronic gastritis patients with or without peptic ulcer. *Eur. J. Immunol.* 27, 1751-1755. 10.1002/eji.1830270723 9247587

[DMM027649C37] den Hoed C. M., van Eijck B. C., Capelle L. G., van Dekken H., Biermann K., Siersema P. D. and Kuipers E. J. (2011). The prevalence of premalignant gastric lesions in asymptomatic patients: predicting the future incidence of gastric cancer. *Eur. J. Cancer* 47, 1211-1218. 10.1016/j.ejca.2010.12.012 21239166

[DMM027649C38] Dicksved J., Lindberg M., Rosenquist M., Enroth H., Jansson J. K. and Engstrand L. (2009). Molecular characterization of the stomach microbiota in patients with gastric cancer and in controls. *J. Med. Microbiol.* 58, 509-516. 10.1099/jmm.0.007302-0 19273648

[DMM027649C39] Drazek E. S., Dubois A. and Holmes R. K. (1994). Characterization and presumptive identification of *Helicobacter pylori* isolates from rhesus monkeys. *J. Clin. Microbiol.* 32, 1799-1804. 7523441 10.1128/jcm.32.7.1799-1804.1994 PMC263799

[DMM027649C40] Dubois A., Fiala N., Heman-Ackah L. M., Drazek E. S., Tarnawski A., Fishbein W. N., Perez-Perez G. I. and Blaser M. J. (1994). Natural gastric infection with *Helicobacter pylori* in monkeys: a model for spiral bacteria infection in humans. *Gastroenterology* 106, 1405-1417. 10.1016/0016-5085(94)90392-1 8194685

[DMM027649C41] Eaton K. A. and Krakowka S. (1994). Effect of gastric pH on urease-dependent colonization of gnotobiotic piglets by *Helicobacter pylori*. *Infect. Immun.* 62, 3604-3607. 8063376 10.1128/iai.62.9.3604-3607.1994 PMC303008

[DMM027649C42] Eaton K. A., Morgan D. R. and Krakowka S. (1992). Motility as a factor in the colonisation of gnotobiotic piglets by *Helicobacter pylori*. *J. Med. Microbiol.* 37, 123-127. 10.1099/00222615-37-2-123 1629897

[DMM027649C43] Eaton K. A., Dewhirst F. E., Radin M. J., Fox J. G., Paster B. J., Krakowka S. and Morgan D. R. (1993). *Helicobacter acinonyx* sp. nov., isolated from cheetahs with gastritis. *Int. J. Syst. Evol. Microbiol.* 43, 99-106. 10.1099/00207713-43-1-99 8379970

[DMM027649C44] Ectors N. and Dixon M. F. (1986). The prognostic value of sulphomucin positive intestinal metaplasia in the development of gastric cancer. *Histopathology* 10, 1271-1277. 10.1111/j.1365-2559.1986.tb02570.x 3817762

[DMM027649C45] Elfvin A., Bölin I., Von Bothmer C., Stolte M., Watanabe H., Fändriks L. and Vieth M. (2005). *Helicobacter pylori* induces gastritis and intestinal metaplasia but no gastric adenocarcinoma in Mongolian gerbils. *Scand. J. Gastroenterol.* 40, 1313-1320. 10.1080/00365520510023611 16334441

[DMM027649C46] Enno A., O'Rourke J. L., Howlett C. R., Jack A., Dixon M. F. and Lee A. (1995). MALToma-like lesions in the murine gastric mucosa after long-term infection with *Helicobacter felis*. A mouse model of *Helicobacter pylori*-induced gastric lymphoma. *Am. J. Pathol.* 147, 217-222. 7604881 PMC1869885

[DMM027649C47] Erdman S. E., Correa P., Coleman L. A., Schrenzel M. D., Li X. and Fox J. G. (1997). *Helicobacter mustelae*-associated gastric MALT lymphoma in ferrets. *Am. J. Pathol.* 151, 273-280. 9212752 PMC1857920

[DMM027649C48] Esmail M. Y., Bacon R., Swennes A. G., Feng Y., Shen Z., Garcia A. P., Sharma P., Cohen J. and Fox J. G. (2016). Helicobacter species identified in captive sooty mangabeys (*Cercocebus atys*) with metastatic gastric adenocarcinoma. *Helicobacter* 21, 175-185. 10.1111/hel.12269 26477442 PMC4837085

[DMM027649C49] Falush D., Wirth T., Linz B., Pritchard J. K., Stephens M., Kidd M., Blaser M. J., Graham D. Y., Vacher S., Perez-Perez G. I. et al. (2003). Traces of human migrations in *Helicobacter pylori* populations. *Science* 299, 1582-1585. 10.1126/science.1080857 12624269

[DMM027649C50] Ferlay J., Soerjomataram I., Ervik M., Dikshit R., Eser S., Mathers C., Rebelo M., Parkin D. M., Forman D. and Bray F. (2013). *GLOBOCAN 2012 v1.0, Cancer Incidence and Mortality Worldwide: IARC CancerBase No. 11 [Internet]*. International Agency for Research on Cancer (http://globocan.iarc.fr).

[DMM027649C51] Filipe M. I., Newell D. G., Johnston B. J., Caygill C. and Reed P. I. (1995). *Helicobacter pylori* in patients with intestinal metaplasia and in controls: a serological and biopsy study in four UK centres. UK Sub-Group of the ECP-EURONUT-Intestinal Metaplasia Study Group. Presented. *Eur. J. Cancer Prevent.* 4, 175-180. 10.1097/00008469-199504000-00008 7767244

[DMM027649C52] Flahou B., Haesebrouck F., Pasmans F., D'Herde K., Driessen A., Van Deun K., Smet A., Duchateau L., Chiers K. and Ducatelle R. (2010). *Helicobacter suis* causes severe gastric pathology in mouse and mongolian gerbil models of human gastric disease. *PLoS ONE* 5, e14083 10.1371/journal.pone.0014083 21124878 PMC2989923

[DMM027649C53] Flahou B., Haesebrouck F., Smet A., Yonezawa H., Osaki T. and Kamiya S. (2013). Gastric and enterohepatic non-*Helicobacter pylori* Helicobacters. *Helicobacter* 18 Suppl. 1, 66-72. 10.1111/hel.12072 24011248

[DMM027649C54] Ford A. C., Forman D., Hunt R. H., Yuan Y. and Moayyedi P. (2014). *Helicobacter pylori* eradication therapy to prevent gastric cancer in healthy asymptomatic infected individuals: systematic review and meta-analysis of randomised controlled trials. *BMJ* 348, g3174 10.1136/bmj.g3174 24846275 PMC4027797

[DMM027649C55] Fox J. G., Cabot E. B., Taylor N. S. and Laraway R. (1988). Gastric colonization by *Campylobacter pylori* subsp. mustelae in ferrets. *Infect. Immun.* 56, 2994-2996. 3169994 10.1128/iai.56.11.2994-2996.1988 PMC259683

[DMM027649C56] Fox J. G., Correa P., Taylor N. S., Lee A., Otto G., Murphy J. C. and Rose R. (1990). *Helicobacter mustelae*-associated gastritis in ferrets. An animal model of *Helicobacter pylori* gastritis in humans. *Gastroenterology* 99, 352-361. 10.1016/0016-5085(90)91016-y 2365188

[DMM027649C57] Fox J. G., Batchelder M., Marini R., Yan L., Handt L., Li X., Shames B., Hayward A., Campbell J. and Murphy J. C. (1995). *Helicobacter pylori*-induced gastritis in the domestic cat. *Infect. Immun.* 63, 2674-2681. 7790084 10.1128/iai.63.7.2674-2681.1995 PMC173358

[DMM027649C58] Fox J. G., Dangler C. A., Sager W., Borkowski R. and Gliatto J. M. (1997). *Helicobacter mustelae*-associated gastric adenocarcinoma in ferrets (*Mustela putorius furo*). *Vet. Pathol.* 34, 225-229. 10.1177/030098589703400308 9163879

[DMM027649C59] Fox J. G., Sheppard B. J., Dangler C. A., Whary M. T., Ihrig M. and Wang T. C. (2002). Germ-Line p53-targeted disruption inhibits Helicobacter-induced premalignant lesions and invasive gastric carcinoma through down-regulation of Th1 proinflammatory responses. *Cancer Res.* 62, 696-702. 11830522

[DMM027649C60] Fox J. G., Rogers A. B., Ihrig M., Taylor N. S., Whary M. T., Dockray G., Varro A. and Wang T. C. (2003). *Helicobacter pylori*-associated gastric cancer in INS-GAS mice is gender specific. *Cancer Res.* 63, 942-950. 12615707

[DMM027649C61] Franco A. T., Israel D. A., Washington M. K., Krishna U., Fox J. G., Rogers A. B., Neish A. S., Collier-Hyams L., Perez-Perez G. I., Hatakeyama M. et al. (2005). Activation of beta-catenin by carcinogenic *Helicobacter pylori*. *Proc. Natl. Acad. Sci. USA* 102, 10646-10651. 10.1073/pnas.0504927102 16027366 PMC1180811

[DMM027649C62] Garcia-Gonzalez M. A., Bujanda L., Quintero E., Santolaria S., Benito R., Strunk M., Sopena F., Thomson C., Perez-Aisa A., Nicolas-Perez D. et al. (2015). Association of PSCA rs2294008 gene variants with poor prognosis and increased susceptibility to gastric cancer and decreased risk of duodenal ulcer disease. *Int. J. Cancer* 137, 1362-1373. 10.1002/ijc.29500 25721731

[DMM027649C63] Gisbert J. P. and Calvet X. (2011). Review article: common misconceptions in the management of *Helicobacter pylori*-associated gastric MALT-lymphoma. *Aliment. Pharmacol. Ther.* 34, 1047-1062. 10.1111/j.1365-2036.2011.04839.x 21919927

[DMM027649C64] Graham D. Y. (1989). *Campylobacter pylori* and peptic ulcer disease. *Gastroenterology* 96, 615-625. 10.1016/S0016-5085(89)80057-5 2642447

[DMM027649C65] Graham D. Y. (2015). *Helicobacter pylori* update: gastric cancer, reliable therapy, and possible benefits. *Gastroenterology* 148, 719-31.e3 10.1053/j.gastro.2015.01.040 PMC4375058 25655557

[DMM027649C66] Graham D. Y., Lu H. and Yamaoka Y. (2009). African, Asian or Indian enigma, the East Asian *Helicobacter pylori*: facts or medical myths. *J. Dig. Dis.* 10, 77-84. 10.1111/j.1751-2980.2009.00368.x 19426388 PMC2846403

[DMM027649C67] Groenen M. J., Kuipers E. J., Hansen B. E. and Ouwendijk R. J. (2009). Incidence of duodenal ulcers and gastric ulcers in a Western population: back to where it started. *Can. J. Gastroenterol.* 23, 604-608. 10.1155/2009/181059 19816622 PMC2776548

[DMM027649C68] Haesebrouck F., Pasmans F., Flahou B., Chiers K., Baele M., Meyns T., Decostere A. and Ducatelle R. (2009). Gastric helicobacters in domestic animals and nonhuman primates and their significance for human health. *Clin. Microbiol. Rev.* 22, 202-223. 10.1128/cmr.00041-08 19366912 PMC2668234

[DMM027649C69] Han S.-U., Kim Y.-B., Joo H.-J., Hahm K.-B., Lee W.-H., Cho Y.-K., Kim D.-Y. and Kim M.-W. (2002). *Helicobacter pylori* infection promotes gastric carcinogenesis in a mice model. *J. Gastroenterol. Hepatol.* 17, 253-261. 10.1046/j.1440-1746.2002.02684.x 11982694

[DMM027649C188] Handt L. K., Fox J. G., Dewhirst F. E., Fraser G. J., Paster B. J., Yan L. L., Rozmiarek H., Rufo R., Stalis I. H. (1994). *Helicobacter pylori* isolated from the domestic cat: public health implications. *Infect Immun.* 62, 2367-2374. 10.1128/JCM.40.12.4536-4543.2002 8188360 PMC186520

[DMM027649C70] Harper C. G., Feng Y., Xu S., Taylor N. S., Kinsel M., Dewhirst F. E., Paster B. J., Greenwell M., Levine G., Rogers A. et al. (2002). *Helicobacter cetorum* sp. nov., a urease-positive Helicobacter species isolated from dolphins and whales. *J. Clin. Microbiol.* 40, 4536-4543. 10.1128/JCM.40.12.4536-4543.2002 12454148 PMC154630

[DMM027649C71] Heilmann K. L. and Borchard F. (1991). Gastritis due to spiral shaped bacteria other than *Helicobacter pylori*: clinical, histological, and ultrastructural findings. *Gut* 32, 137-140. 10.1136/gut.32.2.137 1864530 PMC1378794

[DMM027649C72] Helicobacter and Cancer Collaborative Group. (2001). Gastric cancer and *Helicobacter pylori*: a combined analysis of 12 case control studies nested within prospective cohorts. *Gut* 49, 347-353. 10.1136/gut.49.3.347 11511555 PMC1728434

[DMM027649C73] Higashi H., Tsutsumi R., Muto S., Sugiyama T., Azuma T., Asaka M. and Hatakeyama M. (2002). SHP-2 tyrosine phosphatase as an intracellular target of *Helicobacter pylori* CagA protein. *Science* 295, 683-686. 10.1126/science.1067147 11743164

[DMM027649C74] Holcombe C. (1992). *Helicobacter pylori*: the African enigma. *Gut* 33, 429-431. 10.1136/gut.33.4.429 1582581 PMC1374052

[DMM027649C75] Honda S., Fujioka T., Tokieda M., Gotoh T., Nishizono A. and Nasu M. (1998a). Gastric ulcer, atrophic gastritis, and intestinal metaplasia caused by *Helicobacter pylori* infection in Mongolian gerbils. *Scand. J. Gastroenterol.* 33, 454-460. 10.1080/00365529850171990 9648982

[DMM027649C76] Honda S., Fujioka T., Tokieda M., Satoh R., Nishizono A. and Nasu M. (1998b). Development of *Helicobacter pylori*-induced gastric carcinoma in Mongolian gerbils. *Cancer Res.* 58, 4255-4259. 9766647

[DMM027649C77] Huang J. Y., Sweeney E. G., Sigal M., Zhang H. C., Remington S. J., Cantrell M. A., Kuo C. J., Guillemin K. and Amieva M. R. (2015). Chemodetection and destruction of host urea allows *Helicobacter pylori* to locate the epithelium. *Cell Host Microbe* 18, 147-156. 10.1016/j.chom.2015.07.002 26269952 PMC4593702

[DMM027649C78] Ichikawa H., Sugimoto M., Uotani T., Sahara S., Yamade M., Iwaizumi M., Yamada T., Osawa S., Sugimoto K., Miyajima H. et al. (2015). Influence of prostate stem cell antigen gene polymorphisms on susceptibility to *Helicobacter pylori*-associated diseases: a case-control study. *Helicobacter* 20, 106-113. 10.1111/hel.12183 25582162

[DMM027649C79] Jakszyn P., Bingham S., Pera G., Agudo A., Luben R., Welch A., Boeing H., Del Giudice G., Palli D., Saieva C. et al. (2006). Endogenous versus exogenous exposure to N-nitroso compounds and gastric cancer risk in the European Prospective Investigation into Cancer and Nutrition (EPIC-EURGAST) study. *Carcinogenesis* 27, 1497-1501. 10.1093/carcin/bgl019 16571648

[DMM027649C80] Jiang Y. and Yu Y. (2017). Transgenic and gene knockout mice in gastric cancer research. *Oncotarget* 8, 3696-3710. 10.18632/oncotarget.12467 27713138 PMC5356912

[DMM027649C81] Judd L. M., Alderman B. M., Howlett M., Shulkes A., Dow C., Moverley J., Grail D., Jenkins B. J., Ernst M. and Giraud A. S. (2004). Gastric cancer development in mice lacking the SHP2 binding site on the IL-6 family co-receptor gp130. *Gastroenterology* 126, 196-207. 10.1053/j.gastro.2003.10.066 14699500

[DMM027649C82] Justice M. J. and Dhillon P. (2016). Using the mouse to model human disease: increasing validity and reproducibility. *Dis. Model. Mech.* 9, 101 10.1242/dmm.024547 26839397 PMC4770152

[DMM027649C83] Kato S., Tsukamoto T., Mizoshita T., Tanaka H., Kumagai T., Ota H., Katsuyama T., Asaka M. and Tatematsu M. (2006). High salt diets dose-dependently promote gastric chemical carcinogenesis in *Helicobacter pylori*-infected Mongolian gerbils associated with a shift in mucin production from glandular to surface mucous cells. *Int. J. Cancer* 119, 1558-1566. 10.1002/ijc.21810 16646055

[DMM027649C84] Kaur B., Garg N., Sachdev A. and Kumar B. (2014). Effect of the oral intake of probiotic *Pediococcus acidilactici* BA28 on *Helicobacter pylori* causing peptic ulcer in C57BL/6 mice models. *Appl. Biochem. Biotechnol.* 172, 973-983. 10.1007/s12010-013-0585-4 24122711

[DMM027649C85] Khalil M. O., Morton L. M., Devesa S. S., Check D. P., Curtis R. E., Weisenburger D. D. and Dores G. M. (2014). Incidence of marginal zone lymphoma in the United States, 2001-2009 with a focus on primary anatomic site. *Br. J. Haematol.* 165, 67-77. 10.1111/bjh.12730 24417667 PMC3967856

[DMM027649C86] Kodaman N., Pazos A., Schneider B. G., Piazuelo M. B., Mera R., Sobota R. S., Sicinschi L. A., Shaffer C. L., Romero-Gallo J., de Sablet T. et al. (2014). Human and *Helicobacter pylori* coevolution shapes the risk of gastric disease. *Proc. Natl. Acad. Sci. USA* 111, 1455-1460. 10.1073/pnas.1318093111 24474772 PMC3910595

[DMM027649C87] Konno M., Fujii N., Yokota S., Sato K., Takahashi M., Sato K., Mino E. and Sugiyama T. (2005). Five-year follow-up study of mother-to-child transmission of *Helicobacter pylori* infection detected by a random amplified polymorphic DNA fingerprinting method. *J. Clin. Microbiol.* 43, 2246-2250. 10.1128/JCM.43.5.2246-2250.2005 15872250 PMC1153758

[DMM027649C88] Konno M., Yokota S., Suga T., Takahashi M., Sato K. and Fujii N. (2008). Predominance of mother-to-child transmission of *Helicobacter pylori* infection detected by random amplified polymorphic DNA fingerprinting analysis in Japanese families. *Pediatr. Infect. Dis. J.* 27, 999-1003. 10.1097/INF.0b013e31817d756e 18845980

[DMM027649C89] Krakowka S., Eaton K. A. and Rings D. M. (1995). Occurrence of gastric ulcers in gnotobiotic piglets colonized by *Helicobacter pylori*. *Infect. Immun.* 63, 2352-2355. 7768620 10.1128/iai.63.6.2352-2355.1995 PMC173310

[DMM027649C90] Krakowka S., Rings D. M. and Ellis J. A. (2005). Experimental induction of bacterial gastritis and gastric ulcer disease in gnotobiotic swine inoculated with porcine Helicobacter-like species. *Am. J. Vet. Res.* 66, 945-952. 10.2460/ajvr.2005.66.945 16008214

[DMM027649C91] Kronsteiner B., Bassaganya-Riera J., Philipson C., Viladomiu M., Carbo A., Pedragosa M., Vento S. and Hontecillas R. (2013). *Helicobacter pylori* infection in a pig model is dominated by Th1 and cytotoxic CD8+ T cell responses. *Infect. Immun.* 81, 3803-3813. 10.1128/IAI.00660-13 23897614 PMC3811743

[DMM027649C92] La T. G., Chiaradia G., Gianfagna F., De Lauretis A., Boccia S., Mannocci A. and Ricciardi W. (2009). Smoking status and gastric cancer risk: an updated meta-analysis of case-control studies published in the past ten years. *Tumori* 95, 13-22. 19366050 10.1177/030089160909500103

[DMM027649C93] Lachman L. B., Ozpolat B., Rao X.-M., Graham D. Y. and Osato M. (1997). Development of a murine model of *Helicobacter pylori* infection. *Helicobacter* 2, 78-81. 10.1111/j.1523-5378.1997.tb00062.x 9432332

[DMM027649C94] Laszewicz W., Iwanczak F. and Iwanczak B., Task Force of the Polish Society of Gastroenterology. (2014). Seroprevalence of *Helicobacter pylori* infection in Polish children and adults depending on socioeconomic status and living conditions. *Adv. Med. Sci.* 59, 147-150. 10.1016/j.advms.2014.01.003 24797992

[DMM027649C95] Lee A., O'Rourke J., De Ungria M. C., Robertson B., Daskalopoulos G. and Dixon M. F. (1997). A standardized mouse model of *Helicobacter pylori* infection: introducing the Sydney strain. *Gastroenterology* 112, 1386-1397. 10.1016/S0016-5085(97)70155-0 9098027

[DMM027649C96] Lee I. O., Kim J. H., Choi Y. J., Pillinger M. H., Kim S.-Y., Blaser M. J. and Lee Y. C. (2010). *Helicobacter pylori* CagA Phosphorylation Status Determines the gp130-activated SHP2/ERK and JAK/STAT Signal Transduction Pathways in Gastric Epithelial Cells. *J. Biol. Chem.* 285, 16042-16050. 10.1074/jbc.M110.111054 20348091 PMC2871473

[DMM027649C97] Lefebvre O., Chenard M.-P., Masson R., Linares J., Dierich A., LeMeur M., Wendling C., Tomasetto C., Chambon P. and Rio M.-C. (1996). Gastric mucosa abnormalities and tumorigenesis in mice lacking the pS2 trefoil protein. *Science* 274, 259-262. 10.1126/science.274.5285.259 8824193

[DMM027649C98] Lertpiriyapong K., Whary M. T., Muthupalani S., Lofgren J. L., Gamazon E. R., Feng Y., Ge Z., Wang T. C. and Fox J. G. (2014). Gastric colonisation with a restricted commensal microbiota replicates the promotion of neoplastic lesions by diverse intestinal microbiota in the *Helicobacter pylori* INS-GAS mouse model of gastric carcinogenesis. *Gut* 63, 54-63. 10.1136/gutjnl-2013-305178 23812323 PMC4023484

[DMM027649C99] Li H., Mellgard B. and Helander H. F. (1997). Inoculation of VacA- and CagA- *Helicobacter pylori* delays gastric ulcer healing in the rat. *Scand. J. Gastroenterol.* 32, 439-444. 10.3109/00365529709025078 9175204

[DMM027649C100] Li Z., Zou D., Ma X., Chen J., Shi X., Gong Y., Man X., Gao L., Zhao Y., Wang R. et al. (2010). Epidemiology of peptic ulcer disease: endoscopic results of the systematic investigation of gastrointestinal disease in China. *Am. J. Gastroenterol.* 105, 2570-2577. 10.1038/ajg.2010.324 20736940

[DMM027649C101] Liang J., De Bruyne E., Ducatelle R., Smet A., Haesebrouck F. and Flahou B. (2015). Purification of *Helicobacter suis* strains from biphasic cultures by single colony isolation: influence on strain characteristics. *Helicobacter* 20, 206-216. 10.1111/hel.12192 25582323

[DMM027649C102] Linz B., Balloux F., Moodley Y., Manica A., Liu H., Roumagnac P., Falush D., Stamer C., Prugnolle F., van der Merwe S. W. et al. (2007). An African origin for the intimate association between humans and *Helicobacter pylori*. *Nature* 445, 915-918. 10.1038/nature05562 17287725 PMC1847463

[DMM027649C103] Liu H., Merrell D. S., Semino-Mora C., Goldman M., Rahman A., Mog S. and Dubois A. (2009). Diet synergistically affects *Helicobacter pylori*-induced gastric carcinogenesis in nonhuman primates. *Gastroenterology* 137, 1367-1379.e6. 10.1053/j.gastro.2009.07.041 19622359 PMC2774828

[DMM027649C104] Liu J., He L., Haesebrouck F., Gong Y., Flahou B., Cao Q. and Zhang J. (2015). Prevalence of coinfection with gastric Non-*Helicobacter pylori* Helicobacter (NHPH) species in *Helicobacter pylori*-infected patients suffering from gastric disease in Beijing, China. *Helicobacter* 20, 284-290. 10.1111/hel.12201 25510739

[DMM027649C105] Lofgren J. L., Whary M. T., Ge Z., Muthupalani S., Taylor N. S., Mobley M., Potter A., Varro A., Eibach D., Suerbaum S. et al. (2011). Lack of commensal flora in *Helicobacter pylori*-infected INS-GAS mice reduces gastritis and delays intraepithelial neoplasia. *Gastroenterology* 140, 210-220. 10.1053/j.gastro.2010.09.048 20950613 PMC3006487

[DMM027649C106] Luzza F., Suraci E., Larussa T., Leone I. and Imeneo M. (2014). High exposure, spontaneous clearance, and low incidence of active *Helicobacter pylori* infection: the Sorbo San Basile study. *Helicobacter* 19, 296-305. 10.1111/hel.12133 24758553

[DMM027649C107] Mendez M. A., Pera G., Agudo A., Bueno-de-Mesquita H. B., Palli D., Boeing H., Carneiro F., Berrino F., Sacerdote C., Tumino R. et al. (2007). Cereal fiber intake may reduce risk of gastric adenocarcinomas: the EPIC-EURGAST study. *Int. J. Cancer* 121, 1618-1623. 10.1002/ijc.22896 17582605

[DMM027649C108] Mahe M. M., Aihara E., Schumacher M. A., Zavros Y., Montrose M. H., Helmrath M. A., Sato T. and Shroyer N. F. (2013). Establishment of gastrointestinal epithelial organoids. *Curr. Protoc. Mouse Biol.* 3, 217-240. 10.1002/9780470942390.mo130179 25105065 PMC4120977

[DMM027649C109] Mamishi S., Eshaghi H., Mahmoudi S., Bahador A., Hosseinpour Sadeghi R., Najafi M., Farahmand F., Khodadad A. and Pourakbari B. (2016). Intrafamilial transmission of *Helicobacter pylori*: genotyping of faecal samples. *Br. J. Biomed. Sci.* 73, 38-43. 10.1080/09674845.2016.1150666 27182676

[DMM027649C110] Marshall B. J., Armstrong J. A., McGechie D. B. and Glancy R. J. (1985). Attempt to fulfil Koch's postulates for pyloric Campylobacter. *Med. J. Aust.* 142, 436-439. 3982345 10.5694/j.1326-5377.1985.tb113443.x

[DMM027649C111] Maruta F., Sugiyama A., Ishida K., Ikeno T., Murakami M., Kawasaki S., Ota H., Tatematsu M. and Katsuyama T. (2000). Timing of N-methyl-N-nitrosourea administration affects gastric carcinogenesis in Mongolian gerbils infected with *Helicobacter pylori*. *Cancer Lett.* 160, 99-105. 10.1016/S0304-3835(00)00571-1 11098090

[DMM027649C112] Maruta F., Ota H., Genta R. M., Sugiyama A., Tatematsu M., Katsuyama T. and Kawasaki S. (2001). Role of N-methyl-N-nitrosourea in the induction of intestinal metaplasia and gastric adenocarcinoma in Mongolian gerbils infected with *Helicobacter pylori*. *Scand. J. Gastroenterol.* 36, 283-290. 10.1080/003655201750074591 11305516

[DMM027649C113] McColl K. E. L., Fullarton G. M., Chittajalu R., el Nujumi A. M., MacDonald A. M. I., Dahill S. W. and Hilditch T. E. (1991). Plasma gastrin, daytime intragastric pH, and nocturnal acid output before and at 1 and 7 months after eradication of *Helicobacter pylori* in duodenal ulcer subjects. *Scand. J. Gastroenterol.* 26, 339-346. 10.3109/00365529109025052 1853158

[DMM027649C114] McColl K. E., el-Omar E. M. and Gillen D. (1997). Alterations in gastric physiology in *Helicobacter pylori* infection: causes of different diseases or all epiphenomena? *Ital. J. Gastroenterol. Hepatol.* 29, 459-464. 9494857

[DMM027649C115] McCracken K. W., Cata E. M., Crawford C. M., Sinagoga K. L., Schumacher M., Rockich B. E., Tsai Y.-H., Mayhew C. N., Spence J. R., Zavros Y. et al. (2014). Modelling human development and disease in pluripotent stem-cell-derived gastric organoids. *Nature* 516, 400-404. 10.1038/nature13863 25363776 PMC4270898

[DMM027649C116] McDonald A. M., Sarfati D., Baker M. G. and Blakely T. (2015). Trends in *Helicobacter pylori* infection among Maori, Pacific, and European Birth cohorts in New Zealand. *Helicobacter* 20, 139-145. 10.1111/hel.12186 25403622

[DMM027649C117] McMillan M., MacKay W. G., Williams C. L., Shepherd A. J., Malcolm C. and Weaver L. T. (2011). Intrafamilial genotyping of *Helicobacter pylori* from faecal DNA. *Gastroenterol. Res. Pract.* 2011, 491035 10.1155/2011/491035 21811496 PMC3147127

[DMM027649C118] McNulty C. A., Dent J. C., Curry A., Uff J. S., Ford G. A., Gear M. W. and Wilkinson S. P. (1989). New spiral bacterium in gastric mucosa. *J. Clin. Pathol.* 42, 585-591. 10.1136/jcp.42.6.585 2738164 PMC1141985

[DMM027649C119] Merga Y. J., O'Hara A., Burkitt M. D., Duckworth C. A., Probert C. S., Campbell B. J. and Pritchard D. M. (2016). Importance of the alternative NF-kappaB activation pathway in inflammation-associated gastrointestinal carcinogenesis. *Am. J. Physiol. Gastrointest. Liver Physiol.* 310, G1081-G1090. 10.1152/ajpgi.00026.2016 27102559

[DMM027649C120] Morris A. and Nicholson G. (1987). Ingestion of *Campylobacter pyloridis* causes gastritis and raised fasting gastric pH. *Am. J. Gastroenterol.* 82, 192-199. 3826027

[DMM027649C121] Morris A. J., Ali M. R., Nicholson G. I., Perez-Perez G. I. and Blaser M. J. (1991). Long-term follow-up of voluntary ingestion of *Helicobacter pylori*. *Ann. Intern. Med.* 114, 662-663. 10.7326/0003-4819-114-8-662 2003713

[DMM027649C122] Mou X., Li T., Wang J., Ali Z., Zhang Y., Chen Z., Deng Y., Li S., Su E., Jia Q. et al. (2015). Genetic Variation of BCL2 (rs2279115), NEIL2 (rs804270), LTA (rs909253), PSCA (rs2294008) and PLCE1 (rs3765524, rs10509670) Genes and Their Correlation to Gastric Cancer Risk Based on Universal Tagged Arrays and Fe3O4 Magnetic Nanoparticles. *J. Biomed. Nanotechnol.* 11, 2057-2066. 10.1166/jbn.2015.2113 26554163

[DMM027649C123] Murray M. J., Schusser G. R., Pipers F. S. and Gross S. J. (1996). Factors associated with gastric lesions in Thoroughbred racehorses. *Equine Vet. J.* 28, 368-374. 10.1111/j.2042-3306.1996.tb03107.x 8894534

[DMM027649C124] Musumba C., Jorgensen A., Sutton L., Van Eker D., Moorcroft J., Hopkins M., Pritchard D. M. and Pirmohamed M. (2012). The relative contribution of NSAIDs and *Helicobacter pylori* to the aetiology of endoscopically-diagnosed peptic ulcer disease: observations from a tertiary referral hospital in the UK between 2005 and 2010. *Aliment. Pharmacol. Ther.* 36, 48-56. 10.1111/j.1365-2036.2012.05118.x 22554233

[DMM027649C125] Nakamura Y., Sakagami T., Yamamoto N., Yokota Y., Koizuka H., Hori K., Fukuda Y., Tanida N., Kobayashi T. and Shimoyama T. (2002). *Helicobacter pylori* does not promote N-methyl-N-nitrosourea-induced gastric carcinogenesis in SPF C57BL/6 mice. *Jpn. J. Cancer Res.* 93, 111-116. 10.1111/j.1349-7006.2002.tb01248.x 11856473 PMC5926948

[DMM027649C126] Nakamura M., Murayama S. Y., Serizawa H., Sekiya Y., Eguchi M., Takahashi S., Nishikawa K., Takahashi T., Matsumoto T., Yamada H. et al. (2007). “Candidatus *Helicobacter heilmannii*” from a cynomolgus monkey induces gastric mucosa-associated lymphoid tissue lymphomas in C57BL/6 mice. *Infect. Immun.* 75, 1214-1222. 10.1128/IAI.01459-06 17194807 PMC1828597

[DMM027649C127] Neiger R. and Simpson K. W. (2000). Helicobacter infection in dogs and cats: facts and fiction. *J. Vet. Intern. Med.* 14, 125-133. 10.1111/j.1939-1676.2000.tb02225.x 10772482

[DMM027649C128] Norris C. R., Marks S. L., Eaton K. A., Torabian S. Z., Munn R. J. and Solnick J. V. (1999). Healthy cats are commonly colonized with “*Helicobacter heilmannii*” that is associated with minimal gastritis. *J. Clin. Microbiol.* 37, 189-194. 9854088 10.1128/jcm.37.1.189-194.1999 PMC84203

[DMM027649C129] Nozaki K., Shimizu N., Inada K., Tsukamoto T., Inoue M., Kumagai T., Sugiyama A., Mizoshita T., Kaminishi M. and Tatematsu M. (2002). Synergistic promoting effects of *Helicobacter pylori* infection and high-salt diet on gastric carcinogenesis in Mongolian gerbils. *Jpn. J. Cancer Res.* 93, 1083-1089. 10.1111/j.1349-7006.2002.tb01209.x 12417037 PMC5926881

[DMM027649C130] Ogura K., Maeda S., Nakao M., Watanabe T., Tada M., Kyutoku T., Yoshida H., Shiratori Y. and Omata M. (2000). Virulence factors of *Helicobacter pylori* responsible for gastric diseases in Mongolian gerbil. *J. Exp. Med.* 192, 1601-1610. 10.1084/jem.192.11.1601 11104802 PMC2193104

[DMM027649C131] Ohnishi N., Yuasa H., Tanaka S., Sawa H., Miura M., Matsui A., Higashi H., Musashi M., Iwabuchi K., Suzuki M. et al. (2008). Transgenic expression of *Helicobacter pylori* CagA induces gastrointestinal and hematopoietic neoplasms in mouse. *Proc. Natl. Acad. Sci. USA* 105, 1003-1008. 10.1073/pnas.0711183105 18192401 PMC2242726

[DMM027649C132] O'Rourke J. L., Dixon M. F., Jack A., Enno A. and Lee A. (2004). Gastric B-cell mucosa-associated lymphoid tissue (MALT) lymphoma in an animal model of ‘*Helicobacter heilmannii*’ infection. *J. Pathol.* 203, 896-903. 10.1002/path.1593 15258991

[DMM027649C133] Patterson M. M., Schrenzel M. D., Feng Y. and Fox J. G. (2000). Gastritis and intestinal metaplasia in syrian hamsters infected with *Helicobacter aurati* and two other microaerobes. *Vet. Pathol.* 37, 589-596. 10.1354/vp.37-6-589 11105948

[DMM027649C134] Peleteiro B., Bastos A., Ferro A. and Lunet N. (2014). Prevalence of *Helicobacter pylori* infection worldwide: a systematic review of studies with national coverage. *Dig. Dis. Sci.* 59, 1698-1709. 10.1007/s10620-014-3063-0 24563236

[DMM027649C135] Persson C., Canedo P., Machado J. C., El-Omar E. M. and Forman D. (2011). Polymorphisms in inflammatory response genes and their association with gastric cancer: a HuGE systematic review and meta-analyses. *Am. J. Epidemiol.* 173, 259-270. 10.1093/aje/kwq370 21178102 PMC3105271

[DMM027649C136] Potet F., Florent C., Benhamou E., Cabrieres F., Bommelaer G., Hostein J., Bigard M. A., Bruley De Varannes S., Colombel J. F. and Rampal P. (1993). Chronic gastritis: prevalence in the French population. CIRIG. *Gastroenterol. Clin. Biol.* 17, 103-108. 8500696

[DMM027649C137] Priestnall S. L., Wiinberg B., Spohr A., Neuhaus B., Kuffer M., Wiedmann M. and Simpson K. W. (2004). Evaluation of “*Helicobacter heilmannii*” subtypes in the gastric mucosas of cats and dogs. *J. Clin. Microbiol.* 42, 2144-2151. 10.1128/JCM.42.5.2144-2151.2004 15131182 PMC404595

[DMM027649C138] Rogers A. B., Taylor N. S., Whary M. T., Stefanich E. D., Wang T. C. and Fox J. G. (2005). *Helicobacter pylori* but not high salt induces gastric intraepithelial neoplasia in B6129 mice. *Cancer Res.* 65, 10709-10715. 10.1158/0008-5472.CAN-05-1846 16322215

[DMM027649C139] Roma-Giannikou E., Karameris A., Balatsos B., Panayiotou J., Manika Z., Van-Vliet C., Rokkas T., Skandalis N. and Kattamis C. (2003). Intrafamilial spread of *Helicobacter pylori*: a genetic analysis. *Helicobacter* 8, 15-20. 10.1046/j.1523-5378.2003.00126.x 12603612

[DMM027649C140] Romero-Gallo J., Harris E. J., Krishna U., Washington M. K., Perez-Perez G. I. and Peek R. M. Jr. (2008). Effect of *Helicobacter pylori* eradication on gastric carcinogenesis. *Lab. Invest.* 88, 328-336. 10.1038/labinvest.3700719 18180700 PMC2833422

[DMM027649C141] Rosebeck S., Madden L., Jin X., Gu S., Apel I. J., Appert A., Hamoudi R. A., Noels H., Sagaert X., Van Loo P. et al. (2011). Cleavage of NIK by the API2-MALT1 fusion oncoprotein leads to noncanonical NF-kappaB activation. *Science* 331, 468-472. 10.1126/science.1198946 21273489 PMC3124150

[DMM027649C142] Rossi G., Rossi M., Vitali C. G., Fortuna D., Burroni D., Pancotto L., Capecchi S., Sozzi S., Renzoni G., Braca G. et al. (1999). A conventional beagle dog model for acute and chronic infection with *Helicobacter pylori*. *Infect. Immun.* 67, 3112-3120. 10338528 10.1128/iai.67.6.3112-3120.1999 PMC96629

[DMM027649C143] Saltanova S. D. (2001). [Prevalence of *Helicobacter pylori* among children and juveniles as evidenced by the 13C urea breath test]. *Lik Sprava* 4, 174-176. 11692707

[DMM027649C144] Sanchez Ceballos F., Taxonera Samso C., Garcia Alonso C., Alba Lopez C., Sainz de Los Terreros Soler L. and Diaz-Rubio M. (2007). [Prevalence of *Helicobacter pylori* infection in the healthy population of Madrid (Spain)]. *Rev. Esp. Enferm. Dig.* 99, 497-501. 18052643 10.4321/s1130-01082007000900003

[DMM027649C145] Schlaermann P., Toelle B., Berger H., Schmidt S. C., Glanemann M., Ordemann J., Bartfeld S., Mollenkopf H. J. and Meyer T. F. (2016). A novel human gastric primary cell culture system for modelling *Helicobacter pylori* infection in vitro. *Gut* 65, 202-213. 10.1136/gutjnl-2014-307949 25539675 PMC4752654

[DMM027649C146] Schofield P. N., Ward J. M. and Sundberg J. P. (2016). Show and tell: disclosure and data sharing in experimental pathology. *Dis. Model. Mech.* 9, 601 10.1242/dmm.026054 27483498 PMC4920154

[DMM027649C147] Schrenzel M. D., Witte C. L., Bahl J., Tucker T. A., Fabian N., Greger H., Hollis C., Hsia G., Siltamaki E. and Rideout B. A. (2010). Genetic characterization and epidemiology of helicobacters in non-domestic animals. *Helicobacter* 15, 126-142. 10.1111/j.1523-5378.2009.00744.x 20402815

[DMM027649C148] Schumacher M. A., Feng R., Aihara E., Engevik A. C., Montrose M. H., Ottemann K. M. and Zavros Y. (2015). *Helicobacter pylori*-induced Sonic Hedgehog expression is regulated by NFkappaB pathway activation: the use of a novel in vitro model to study epithelial response to infection. *Helicobacter* 20, 19-28. 10.1111/hel.12152 25495001 PMC4871133

[DMM027649C149] Seel D. J., Kawabata T., Nakamura M., Ishibashi T., Hamano M., Mashimo M., Shin S. H., Sakamoto K., Jhee E. C. and Watanabe S. (1994). N-nitroso compounds in two nitrosated food products in southwest Korea. *Food Chem. Toxicol.* 32, 1117-1123. 10.1016/0278-6915(94)90127-9 7813983

[DMM027649C150] Sharma P., Cohen J. K., Paul K. S., Courtney C. L., Johnson Z. P. and Anderson D. C. (2011). Spontaneous gastric carcinomas in sooty mangabeys (*Cercocebus atys*). *Comp. Med.* 61, 527-531. 22330580 PMC3236695

[DMM027649C151] Shimada M., Ina K., Kyokane K., Imada A., Yamaguchi H., Nishio Y., Hayakawa M., Iinuma Y., Ohta M., Ando T. et al. (2002). Upregulation of mucosal soluble fas ligand and interferon-gamma may be involved in ulcerogenesis in patients with *Helicobacter pylori*-positive gastric ulcer. *Scand. J. Gastroenterol.* 37, 501-511. 10.1080/00365520252903026 12059049

[DMM027649C152] Shimizu T., Choi E., Petersen C. P., Noto J. M., Romero-Gallo J., Piazuelo M. B., Washington M. K., Peek R. M. Jr. and Goldenring J. R. (2016). Characterization of progressive metaplasia in the gastric corpus mucosa of Mongolian gerbils infected with *Helicobacter pylori*. *J. Pathol.* 239, 399-410. 10.1002/path.4735 27125972 PMC4958595

[DMM027649C153] Shiratori S., Mabe K., Yoshii S., Takakuwa Y., Sato M., Nakamura M., Kudo T., Kato M., Asaka M. and Sakamoto N. (2016). Two cases of chronic gastritis with *non-Helicobacter pylori Helicobacter* infection. *Int. Med.* 55, 1865-1869. 10.2169/internalmedicine.55.5891 27432094

[DMM027649C154] Sigal M., Rothenberg M. E., Logan C. Y., Lee J. Y., Honaker R. W., Cooper R. L., Passarelli B., Camorlinga M., Bouley D. M., Alvarez G. et al. (2015). *Helicobacter pylori* activates and expands Lgr5(+) stem cells through direct colonization of the gastric glands. *Gastroenterology* 148, 1392-1404.e21. 10.1053/j.gastro.2015.02.049 25725293

[DMM027649C155] Silberg D. G., Sullivan J., Kang E., Swain G. P., Moffett J., Sund N. J., Sackett S. D. and Kaestner K. H. (2002). Cdx2 ectopic expression induces gastric intestinal metaplasia in transgenic mice. *Gastroenterology* 122, 689-696. 10.1053/gast.2002.31902 11875002

[DMM027649C156] Slomiany B. L., Piotrowski J. and Slomiany A. (1998). Induction of caspase-3 and nitric oxide synthase-2 during gastric mucosal inflammatory reaction to *Helicobacter pylori* lipopolysaccharide. *IUBMB Life* 46, 1063-1070. 10.1080/15216549800204612 9861460

[DMM027649C157] Snider J. L., Allison C., Bellaire B. H., Ferrero R. L. and Cardelli J. A. (2008). The beta1 integrin activates JNK independent of CagA, and JNK activation is required for *Helicobacter pylori* CagA+-induced motility of gastric cancer cells. *J. Biol. Chem.* 283, 13952-13963. 10.1074/jbc.M800289200 18356158 PMC2376240

[DMM027649C158] Sobala G. M., Crabtree J. E., Dixon M. F., Schorah C. J., Taylor J. D., Rathbone B. J., Heatley R. V. and Axon A. T. (1991). Acute *Helicobacter pylori* infection: clinical features, local and systemic immune response, gastric mucosal histology, and gastric juice ascorbic acid concentrations. *Gut* 32, 1415-1418. 10.1136/gut.32.11.1415 1752479 PMC1379180

[DMM027649C159] Solnick J. V., Chang K., Canfield D. R. and Parsonnet J. (2003). Natural acquisition of *Helicobacter pylori* infection in newborn rhesus macaques. *J. Clin. Microbiol.* 41, 5511-5516. 10.1128/JCM.41.12.5511-5516.2003 14662932 PMC309038

[DMM027649C160] Solnick J. V., Fong J., Hansen L. M., Chang K., Canfield D. R. and Parsonnet J. (2006). Acquisition of *Helicobacter pylori* infection in rhesus macaques is most consistent with oral-oral transmission. *J. Clin. Microbiol.* 44, 3799-3803. 10.1128/JCM.01482-06 17021115 PMC1594807

[DMM027649C161] Stolte M., Bayerdorffer E., Morgner A., Alpen B., Wundisch T., Thiede C. and Neubauer A. (2002). Helicobacter and gastric MALT lymphoma. *Gut* 50 Suppl. 3, iii19-iii24. 10.1136/gut.50.suppl_3.iii19 11953328 PMC1867678

[DMM027649C162] Straubinger R. K., Greiter A., McDonough S. P., Gerold A., Scanziani E., Soldati S., Dailidiene D., Dailide G., Berg D. E. and Simpson K. W. (2003). Quantitative evaluation of inflammatory and immune responses in the early stages of chronic *Helicobacter pylori* infection. *Infect. Immun.* 71, 2693-2703. 10.1128/IAI.71.5.2693-2703.2003 12704144 PMC153233

[DMM027649C163] Suerbaum S. and Josenhans C. (2007). *Helicobacter pylori* evolution and phenotypic diversification in a changing host. *Nat. Rev. Microbiol.* 5, 441-452. 10.1038/nrmicro1658 17505524

[DMM027649C164] Sung J. J. Y., Kuipers E. J. and El-Serag H. B. (2009). Systematic review: the global incidence and prevalence of peptic ulcer disease. *Aliment. Pharmacol. Ther.* 29, 938-946. 10.1111/j.1365-2036.2009.03960.x 19220208

[DMM027649C165] Tang Y., Zhu J., Chen L., Zhang S. and Lin J. (2008). Associations of matrix metalloproteinase-9 protein polymorphisms with lymph node metastasis but not invasion of gastric cancer. *Clin. Cancer Res.* 14, 2870-2877. 10.1158/1078-0432.CCR-07-4042 18451255

[DMM027649C166] Tatematsu M., Yamamoto M., Shimizu N., Yoshikawa A., Fukami H., Kaminishi M., Oohara T., Sugiyama A. and Ikeno T. (1998). Induction of glandular stomach cancers in *Helicobacter pylori*-sensitive Mongolian gerbils treated with N-methyl-N-nitrosourea and N-methyl-N'-nitro-N-nitrosoguanidine in drinking water. *Jpn. J. Cancer Res.* 89, 97-104. 10.1111/j.1349-7006.1998.tb00535.x 9548434 PMC5921771

[DMM027649C167] Taulescu M. A., Valentine B. A., Amorim I., Gartner F., Dumitrascu D. L., Gal A. F., Sevastre B. and Catoi C. (2014). Histopathological features of canine spontaneous non-neoplastic gastric polyps - a retrospective study of 15 cases. *Histol. Histopathol.* 29, 65-75. 10.14670/HH-29.65 23821543

[DMM027649C168] Tebbutt N. C., Giraud A. S., Inglese M., Jenkins B., Waring P., Clay F. J., Malki S., Alderman B. M., Grail D., Hollande F. et al. (2002). Reciprocal regulation of gastrointestinal homeostasis by SHP2 and STAT-mediated trefoil gene activation in gp130 mutant mice. *Nat. Med.* 8, 1089-1097. 10.1038/nm763 12219085

[DMM027649C169] Terio K. A., Munson L., Marker L., Aldridge B. M. and Solnick J. V. (2005). Comparison of Helicobacter spp. in Cheetahs (*Acinonyx jubatus*) with and without Gastritis. *J. Clin. Microbiol.* 43, 229-234. 10.1128/JCM.43.1.229-234.2005 15634976 PMC540127

[DMM027649C170] Thompson J., Epting T., Schwarzkopf G., Singhofen A., Eades-Perner A.-M., van Der Putten H. and Zimmermann W. (2000). A transgenic mouse line that develops early-onset invasive gastric carcinoma provides a model for carcinoembryonic antigen-targeted tumor therapy. *Int. J. Cancer* 86, 863-869. 10.1002/(SICI)1097-0215(20000615)86:6<863::AID-IJC16%3.0.CO;2-4 10842202

[DMM027649C171] Tsuzuki T., Egashira A., Igarashi H., Iwakuma T., Nakatsuru Y., Tominaga Y., Kawate H., Nakao K., Nakamura K., Ide F. et al. (2001). Spontaneous tumorigenesis in mice defective in the MTH1 gene encoding 8-oxo-dGTPase. *Proc. Natl. Acad. Sci. USA* 98, 11456-11461. 10.1073/pnas.191086798 11572992 PMC58751

[DMM027649C172] Tu S., Bhagat G., Cui G., Takaishi S., Kurt-Jones E. A., Rickman B., Betz K. S., Penz-Oesterreicher M., Bjorkdahl O., Fox J. G. et al. (2008). Overexpression of interleukin-1beta induces gastric inflammation and cancer and mobilizes myeloid-derived suppressor cells in mice. *Cancer Cell* 14, 408-419. 10.1016/j.ccr.2008.10.011 18977329 PMC2586894

[DMM027649C173] van Blankenstein M., van Vuuren A. J., Looman C. W. N., Ouwendijk M. and Kuipers E. J. (2013). The prevalence of *Helicobacter pylori* infection in the Netherlands. *Scand. J. Gastroenterol.* 48, 794-800. 10.3109/00365521.2013.799221 23795659

[DMM027649C174] Van den Bulck K., Decostere A., Baele M., Driessen A., Debongnie J.-C., Burette A., Stolte M., Ducatelle R. and Haesebrouck F. (2005). Identification of non-*Helicobacter pylori* spiral organisms in gastric samples from humans, dogs, and cats. *J. Clin. Microbiol.* 43, 2256-2260. 10.1128/JCM.43.5.2256-2260.2005 15872252 PMC1153784

[DMM027649C175] Van den Bulck K., Decostere A., Baele M., Vandamme P., Mast J., Ducatelle R. and Haesebrouck F. (2006). Helicobacter cynogastricus sp. nov., isolated from the canine gastric mucosa. *Int. J. Syst. Evol. Microbiol.* 56, 1559-1564. 10.1099/ijs.0.63860-0 16825630

[DMM027649C176] VanDussen K. L., Marinshaw J. M., Shaikh N., Miyoshi H., Moon C., Tarr P. I., Ciorba M. A. and Stappenbeck T. S. (2015). Development of an enhanced human gastrointestinal epithelial culture system to facilitate patient-based assays. *Gut* 64, 911-920. 10.1136/gutjnl-2013-306651 25007816 PMC4305344

[DMM027649C177] Wang T. C. and Brand S. J. (1992). Function and regulation of gastrin in transgenic mice: a review. *Yale J. Biol. Med.* 65, 705-713; discussion 737-740. 1341073 PMC2589775

[DMM027649C178] Wang T. C., Goldenring J. R., Dangler C., Ito S., Mueller A., Jeon W. K., Koh T. J. and Fox J. G. (1998). Mice lacking secretory phospholipase A2 show altered apoptosis and differentiation with Helicobacter felis infection. *Gastroenterology* 114, 675-689. 10.1016/S0016-5085(98)70581-5 9516388

[DMM027649C179] Wang T. C., Dangler C. A., Chen D., Goldenring J. R., Koh T., Raychowdhury R., Coffey R. J., Ito S., Varro A., Dockray G. J. et al. (2000). Synergistic interaction between hypergastrinemia and Helicobacter infection in a mouse model of gastric cancer. *Gastroenterology* 118, 36-47. 10.1016/S0016-5085(00)70412-4 10611152

[DMM027649C180] Wang X.-Q., Terry P. D. and Yan H. (2009). Review of salt consumption and stomach cancer risk: epidemiological and biological evidence. *World J.Gastroenterol.* 15, 2204-2213. 10.3748/wjg.15.2204 19437559 PMC2682234

[DMM027649C181] Watanabe T., Tada M., Nagai H., Sasaki S. and Nakao M. (1998). *Helicobacter pylori* infection induces gastric cancer in mongolian gerbils. *Gastroenterology* 115, 642-648. 10.1016/S0016-5085(98)70143-X 9721161

[DMM027649C182] Weis V. G. and Goldenring J. R. (2009). Current understanding of SPEM and its standing in the preneoplastic process. *Gastric Cancer* 12, 189-197. 10.1007/s10120-009-0527-6 20047123 PMC4502916

[DMM027649C183] Whary M. T., Muthupalani S., Ge Z., Feng Y., Lofgren J., Shi H. N., Taylor N. S., Correa P., Versalovic J., Wang T. C. et al. (2014). Helminth co-infection in *Helicobacter pylori* infected INS-GAS mice attenuates gastric premalignant lesions of epithelial dysplasia and glandular atrophy and preserves colonization resistance of the stomach to lower bowel microbiota. *Microbes Infect.* 16, 345-355. 10.1016/j.micinf.2014.01.005 24513446 PMC4030519

[DMM027649C184] Yamaoka Y. (2010). Mechanisms of disease: *Helicobacter pylori* virulence factors. *Nat. Rev. Gastroenterol. Hepatol.* 7, 629-641. 10.1038/nrgastro.2010.154 20938460 PMC3137895

[DMM027649C185] Yoshizawa N., Takenaka Y., Yamaguchi H., Tetsuya T., Tanaka H., Tatematsu M., Nomura S., Goldenring J. R. and Kaminishi M. (2007). Emergence of spasmolytic polypeptide-expressing metaplasia in Mongolian gerbils infected with *Helicobacter pylori*. *Lab. Invest.* 87, 1265-1276. 10.1038/labinvest.3700682 18004396

[DMM027649C186] You W.-C., Li J.-Y., Blot W. J., Chang Y.-S., Jin M.-L., Gail M. H., Zhang L., Liu W.-D., Ma J.-L., Hu Y.-R. et al. (1999). Evolution of precancerous lesions in a rural Chinese population at high risk of gastric cancer. *Int. J. Cancer* 83, 615-619. 10.1002/(SICI)1097-0215(19991126)83:5<615::AID-IJC8%3.0.CO;2-L 10521796

[DMM027649C187] Zhang J., Zhang F. and Niu R. (2015). Functions of Shp2 in cancer. *J. Cell Mol. Med.* 19, 2075-2083. 10.1111/jcmm.12618 26088100 PMC4568912

